# Trabecular-Like Scaffold Dictates Osteogenesis via Fluid Shear Stress-Induced Metabolic Reprogramming through the CAV1–HIF-1α Axis

**DOI:** 10.34133/research.1307

**Published:** 2026-06-16

**Authors:** Wang Gong, Jing Zhang, Xinyun Liu, Sheng Zhou, Keke Huang, Jing Jin, Jiasen Gu, Jie Cui, Ao Li, Huixin Liang, Peng Wang, Qing Jiang

**Affiliations:** ^1^ State Key Laboratory of Pharmaceutical Biotechnology, Division of Sports Medicine and Adult Reconstructive Surgery, Department of Orthopedic Surgery, Nanjing Drum Tower Hospital, The Affiliated Hospital of Nanjing University Medical School, Nanjing 210008, China.; ^2^ Branch of National Clinical Research Center for Orthopedics, Sports Medicine and Rehabilitation, Nanjing 210008, China.; ^3^ Co-Innovation Center of Neuroregeneration, Nantong University, Nantong 226019, China.; ^4^ School of Stomatology, Xuzhou Medical University, Xuzhou 221004, China.; ^5^ College of Mechanical and Electrical Engineering, Nanjing University of Aeronautics and Astronautics, Nanjing 210016, China.; ^6^ Department of Otolaryngology Head and Neck Surgery, Jiangsu Provincial Key Medical Discipline (Laboratory), Nanjing Drum Tower Hospital, Affiliated Hospital of Medical School, Nanjing University, Nanjing, China.

## Abstract

The structural design of bone scaffolds determines the local fluid mechanical microenvironment; however, how such cues program stem cell metabolism to drive osteogenesis remains unclear. In this study, Voronoi-based trabecular-like scaffolds with tunable porosity were engineered to modulate fluid shear stress (FSS) while preserving a consistent topology. Computational fluid dynamics analyses confirmed that architectures with lower porosity generated higher FSS, enabling controlled investigation of mechano-metabolic coupling. Under dynamic culture conditions, bone marrow mesenchymal stem cells (BMSCs) cultured on high-FSS scaffolds exhibited enhanced osteogenic differentiation in vitro and promoted bone regeneration in vivo. Integrated transcriptomic, proteomic, and metabolomic analyses identified caveolin-1 (CAV1) as a prominent FSS-responsive membrane regulator. Mechanistically, CAV1 enhanced phosphatidylinositol 3-kinase (PI3K)–AKT signaling, stabilized hypoxia-inducible factor-1α (HIF-1α), and induced a glycolytic shift that supports the energetic and biosynthetic demands of osteogenesis. Pharmacological inhibition of PI3K, HIF-1α, or glycolysis abolished FSS-driven osteogenic responses, validating a CAV1-centered mechano-metabolic axis. These findings establish a direct link between scaffold microarchitecture and metabolic regulation of osteogenesis and provide design principles for mechanically instructive bone repair materials.

## Introduction

Large bone reconstruction remains a major clinical challenge associated with multiple limitations, and scaffolds with biomimetic structures represent one of the most promising strategies for bone filling and regeneration [1–4]. In the development of conventional 3-dimensional (3D)-printed scaffolds, mechanical compatibility with native bone tissue and the capacity to regulate the osteogenic healing process are central considerations [5–7]. Bone regeneration relies on the coordinated integration of cellular metabolism and the surrounding biophysical microenvironment [8]. Although various biochemical factors contribute to this process, mechanical cues play a critical role in directing stem cell metabolism [9]. Existing studies have primarily focused on tensile or compressive loading [10,11]. However, the lacunar–canalicular system of bone also exhibits a distinctive fluid mechanical environment [12,13]. Fluid shear stress (FSS) has been identified as a key physical stimulus regulating the osteogenic differentiation of BMSCs [14,15]. Owing to the complexity of replicating the native bone microenvironment in vitro, the mechanisms by which FSS regulates osteogenic differentiation remain insufficiently understood [16].

The biomimetic design of scaffolds is largely reflected in their ability to simulate fluid dynamic behavior during bone regeneration through diverse pore architectures [17]. Structural parameters, including pore shape, pore size, and porosity, directly influence the internal fluid mechanical microenvironment, particularly the magnitude and spatial distribution of FSS. Random porous structures with incomplete connectivity produced by freeze-drying often generate uncontrollable flow fields, whereas simple lattice or isotropic foam architectures differ substantially from the complex porous networks of native cancellous bone [18]. Voronoi-based design provides a robust framework for constructing scaffolds with irregular and interconnected pore geometries that closely resemble trabecular bone [19]. These parameterized networks facilitate cell infiltration and provide predictable pathways for physiological fluid transport. Importantly, Voronoi-based models accurately replicate the random, normal-distributed pore characteristics of native cancellous bone [20]. By maintaining a consistent spatial topological skeleton, these models allow for the precise modulation of porosity strictly through the adjustment of strut thickness. Consequently, variations in porosity inherently alter the average pore size, faithfully mimicking the physiological coupling of trabecular thinning or thickening. This structural evolution effectively modulates the fluid-mechanical environment, particularly FSS, providing an excellent platform to investigate architecture-driven mechanobiology. This methodological advantage offers a unique platform to isolate and investigate the specific role of FSS in regulating cell fate.

Within the bone marrow cavity, BMSCs perceive mechanical cues through surface receptors, cytoskeletal reorganization, and remodeling of membrane microdomains. Mechanical stimulation activates signaling pathways such as mitogen-activated protein kinase (MAPK) and Yes-associated protein/transcriptional coactivator with PDZ-binding motif (YAP/TAZ), thereby promoting osteogenic differentiation [21–23]. However, how FSS is specifically sensed and translated into molecular instructions that initiate osteogenesis remains unclear. Osteogenic differentiation is an energetically demanding process [24]. During the early stages of osteogenesis, BMSCs preferentially rely on aerobic glycolysis, which rapidly generates adenosine triphosphate (ATP) and provides biosynthetic intermediates required for extracellular matrix (ECM) formation [25,26]. Although the importance of mechanotransduction and cellular metabolism in bone regeneration is widely recognized, the interaction between mechanical stimulation and metabolic reprogramming is likely essential for understanding how mechanical forces govern osteogenic differentiation.

The present study aimed to systematically elucidate the crosstalk between mechanotransduction and cellular metabolism. By constructing Voronoi-based scaffolds with different porosities, precise and stable modulation of the fluid mechanical microenvironment was achieved, allowing investigation of the relationship between FSS and osteogenic differentiation. Higher FSS substantially up-regulated caveolin-1 (CAV1), which is the principal structural protein of caveolae and a key sensor of membrane curvature and mechanical tension [27,28]. Enhanced receptor clustering subsequently activated phosphatidylinositol 3-kinase (PI3K)–AKT signaling and stabilized hypoxia-inducible factor-1α (HIF-1α), thereby promoting a metabolic shift toward glycolysis. Through mechanical simulations, multi-omics analyses, and experimental validation, this study demonstrates that the CAV1–HIF-1α axis plays a central role in transducing FSS into glycolytic activation, which initiates osteogenic differentiation. These findings provide a mechanistic foundation for the rational design of next-generation bone scaffolds.

## Results

### Mechanical and permeability characteristics

**Fig. 1.**
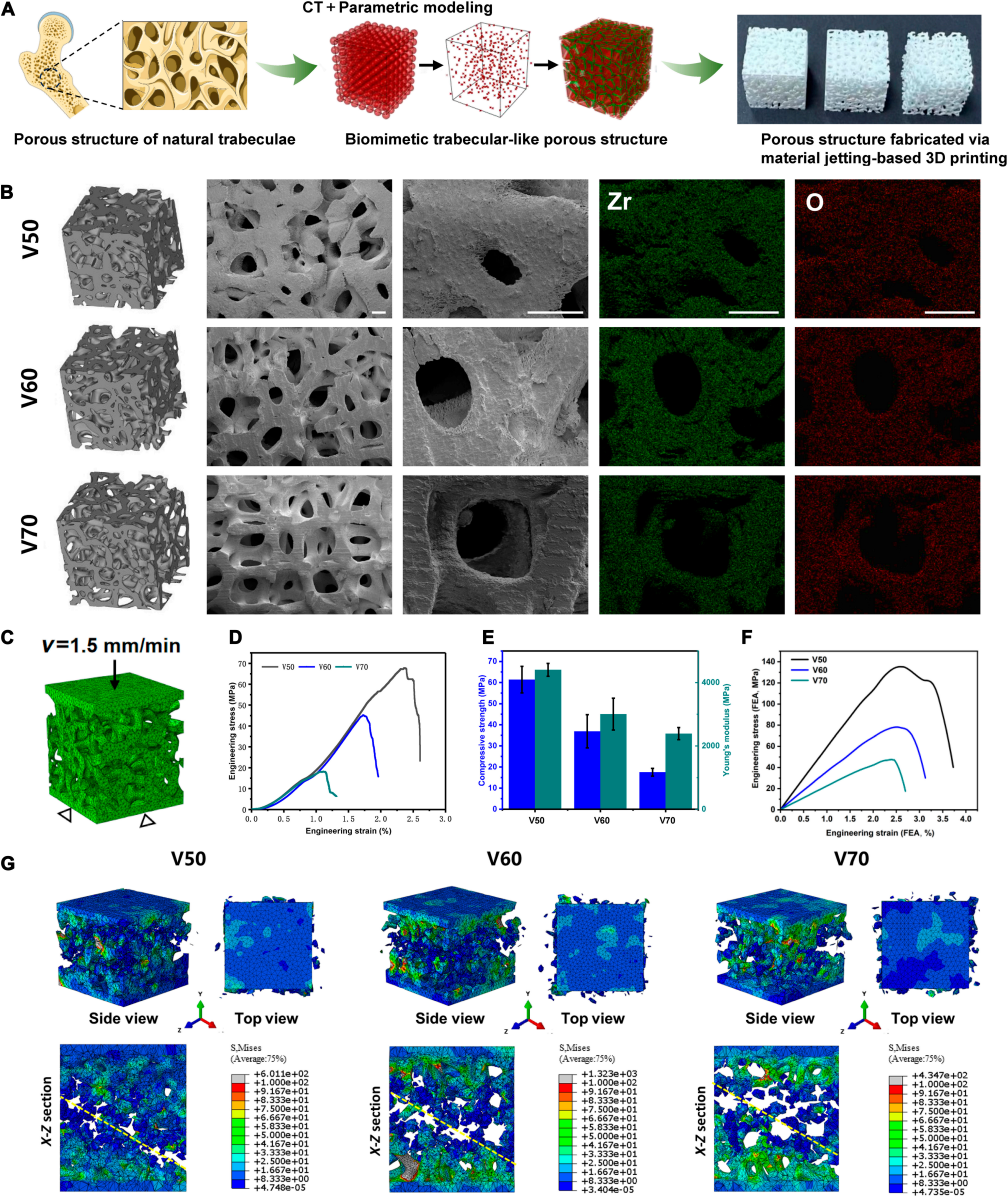
Fabrication, microstructural characterization, and mechanical evaluation of biomimetic Voronoi-based trabecular-like scaffolds. (A) Schematic illustration of the fabrication process of biomimetic Voronoi-based trabecular-like scaffolds via material jetting. (B) Structural models, corresponding scanning electron microscopy images, and elemental mapping by energy-dispersive x-ray spectroscopy demonstrating microstructural variations among scaffolds. Scale bars, 500 μm. (C) Schematic of the experimental setup for compressive modulus testing. (D) Quasi-static compressive stress–strain curves of the different scaffolds. (E) Comparison of compressive strength and Young’s modulus among the scaffold designs. (F) FEA-derived stress–strain curves of the various scaffolds. (G) FEA showing stress distribution nephograms under compressive loading.

The trabecular-inspired porous architectures were designed using a Voronoi diagram combined with a probability ball algorithm, an irregular biomimetic modeling approach established in our previous work. The samples were fabricated by material jetting-based 3D printing using zirconia, a bioinert ceramic, as the feedstock, and the fabrication process is schematically illustrated in Fig. 1A. Three types of Voronoi-based scaffolds, designated as V50, V60, and V70, were generated with actual porosities of 55%, 65%, and 75%, respectively. This naming convention was adopted for simplicity and readability, reflecting porosity ranges rather than exact values. These porosity levels were selected to mimic the structural characteristics of native cancellous bone, which typically exhibits a highly porous architecture with physiological porosity ranging from approximately 50% to 90% and an elastic modulus of 0.1 to 2 GPa. Therefore, the chosen range (55% to 75%) represents the typical density and mechanical properties of healthy human trabecular bone. Environmental scanning electron microscopy coupled with energy-dispersive spectroscopy (ESEM-EDS) analysis of the sintered scaffolds confirmed faithful replication of trabecular bone-like microarchitecture, characterized by homogeneous material distribution and well-interconnected porous networks. The pore size increased progressively with increasing porosity, consistent with the design parameters (Fig. 1B). Quasi-static compression tests were conducted at a constant crosshead displacement rate of 1.5 mm/min until structural failure, as schematically illustrated in Fig. 1C. The porous zirconia trabecular-like scaffold exhibited a relatively short plateau region during compression, which can be attributed to the intrinsic brittleness of the material. As porosity increased from 55% to 65% and 75%, both the yield strength and elastic modulus of the scaffold gradually decreased. This trend was consistent with the classical Gibson–Ashby model for porous materials (Fig. 1D and E). By curve fitting, quantitative relationships between porosity and yield strength or elastic modulus were established for the porous zirconia trabecular-like scaffold, as described by Eqs. (1) and (2):

$$\sigma =360.6{\left(1-\frac{\Phi }{100}\right)}^{2.22}$$ (1)

$$E=10,137{\left(1-\frac{\Phi }{100}\right)}^{1.05}$$ (2)

Finite element simulation results demonstrated that the predicted stress–strain curve exhibited a trend similar to the experimental data (Fig. 1F). During compression, the fracture plane of the scaffold formed an angle of approximately 40° to 60° relative to the compression axis. This feature became more pronounced at lower porosities (Fig. 1G), indicating improved mechanical stability within a specific porosity range. By modulating porosity, the strength and elastic modulus of the scaffold can therefore be tailored to more closely match those of natural bone.

**Fig. 2.**
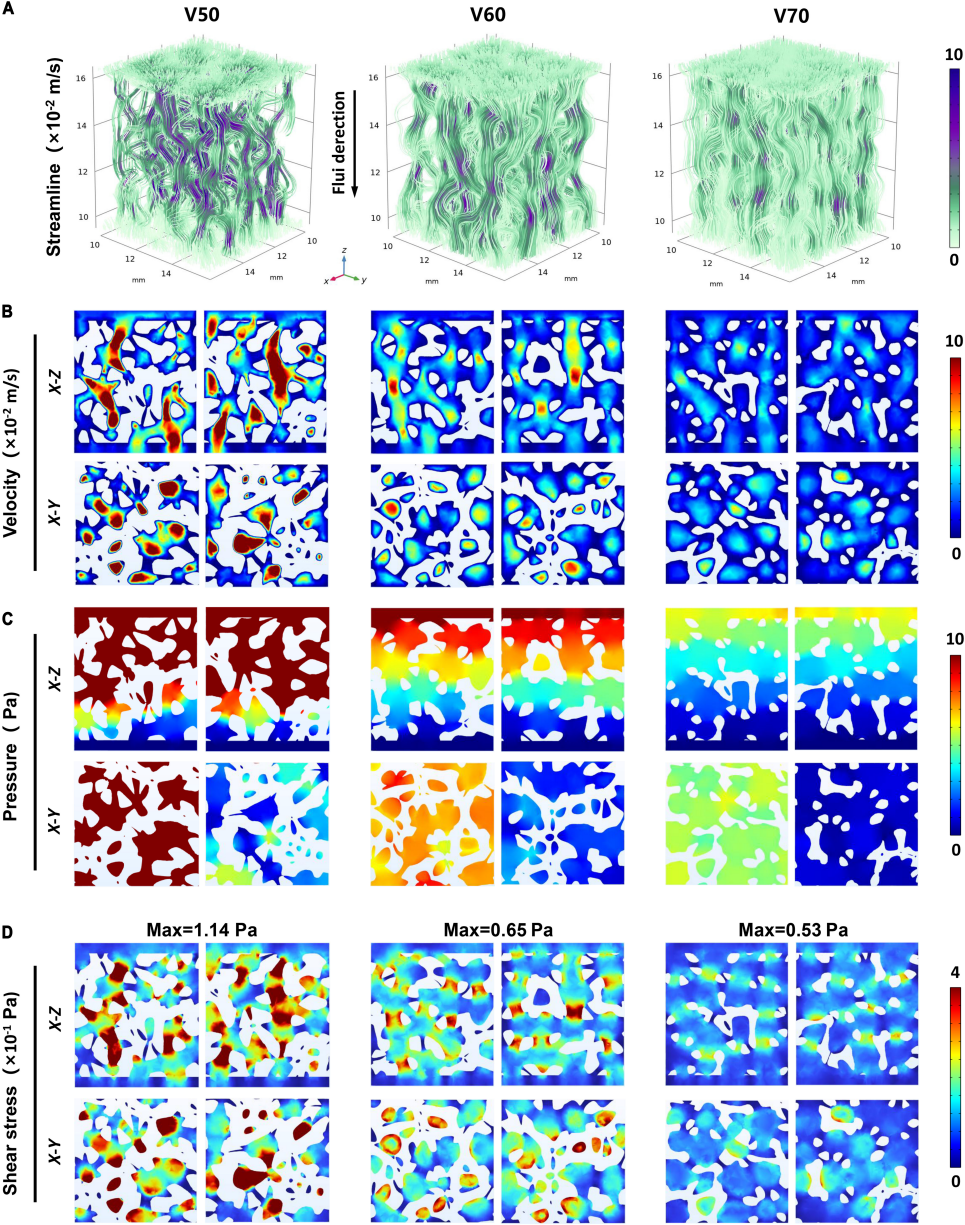
CFD analysis of internal flow characteristics in Voronoi-based trabecular-like scaffolds. (A) 3D velocity fields visualized with streamlines. (B) Cross-sectional velocity fields at selected axial positions. (C) Cross-sectional pressure distributions at corresponding positions. (D) Cross-sectional shear stress fields at the same axial levels.

According to fluid dynamics simulations, the outlet velocity of the porous zirconia trabecular-like scaffold remained largely unchanged with increasing porosity, whereas the pressure drop decreased gradually, resulting in enhanced permeability (Table 1 and Fig. 2C). The scaffold with 55% porosity exhibited a permeability of 2.07 × 10^−9^ m^2^, which was comparable to that of natural bone (0.9 × 10^−9^ m^2^). Streamline profiles (Fig. 2A) revealed that the irregular yet fully interconnected trabecular-like architecture induced multidirectional flow redirection. Consequently, a wide distribution of fluid velocities and surface shear stresses was observed across planes parallel to the flow direction (*X*–*Z* plane) and perpendicular to it (*X*–*Y* plane) (Fig. 2B and D). Such heterogeneity may provide a spatially diverse and favorable mechanical stimulation environment for osteogenesis [17]. However, this variability diminished as porosity increased. Within a certain range, therefore, a lower porosity, such as 55%, may enhance material exchange between tissues and body fluids, thereby potentially promoting new bone ingrowth.

**Table 1.** Permeability parameters

	Porosity (%)	Pressure drop (10^3^ N/m^3^)	Superficial outlet velocity (×10^−3^ m/s)	Permeability (×10^−9^ m^2^)
V50	55	4.73	9.77	2.07
V60	65	1.63	9.82	6.12
V70	75	0.95	9.80	10.3

### Voronoi scaffolds with low porosity provide a favorable microenvironment for BMSC survival and osteogenic differentiation under dynamic FSS

To first establish whether dynamic mechanical stimulation itself enhances osteogenic differentiation in our scaffold-based culture system, primary mouse BMSCs were cultured under static or high FSS (HFSS) conditions and then subjected to osteogenic induction. Relative to the static group, HFSS increased the protein expression of osteogenic markers, including COL1A1, RUNX2, ALP, and OPN (Fig. S1A and B). Quantitative polymerase chain reaction (qPCR) analysis showed a consistent increase in osteogenesis-related gene expression in the HFSS group (Fig. S1C), which was further supported by stronger ALP staining after osteogenic induction (Fig. S1D). These data support that HFSS, even within the same scaffold background, enhances the osteogenic response of mouse BMSCs.

**Fig. 3.**
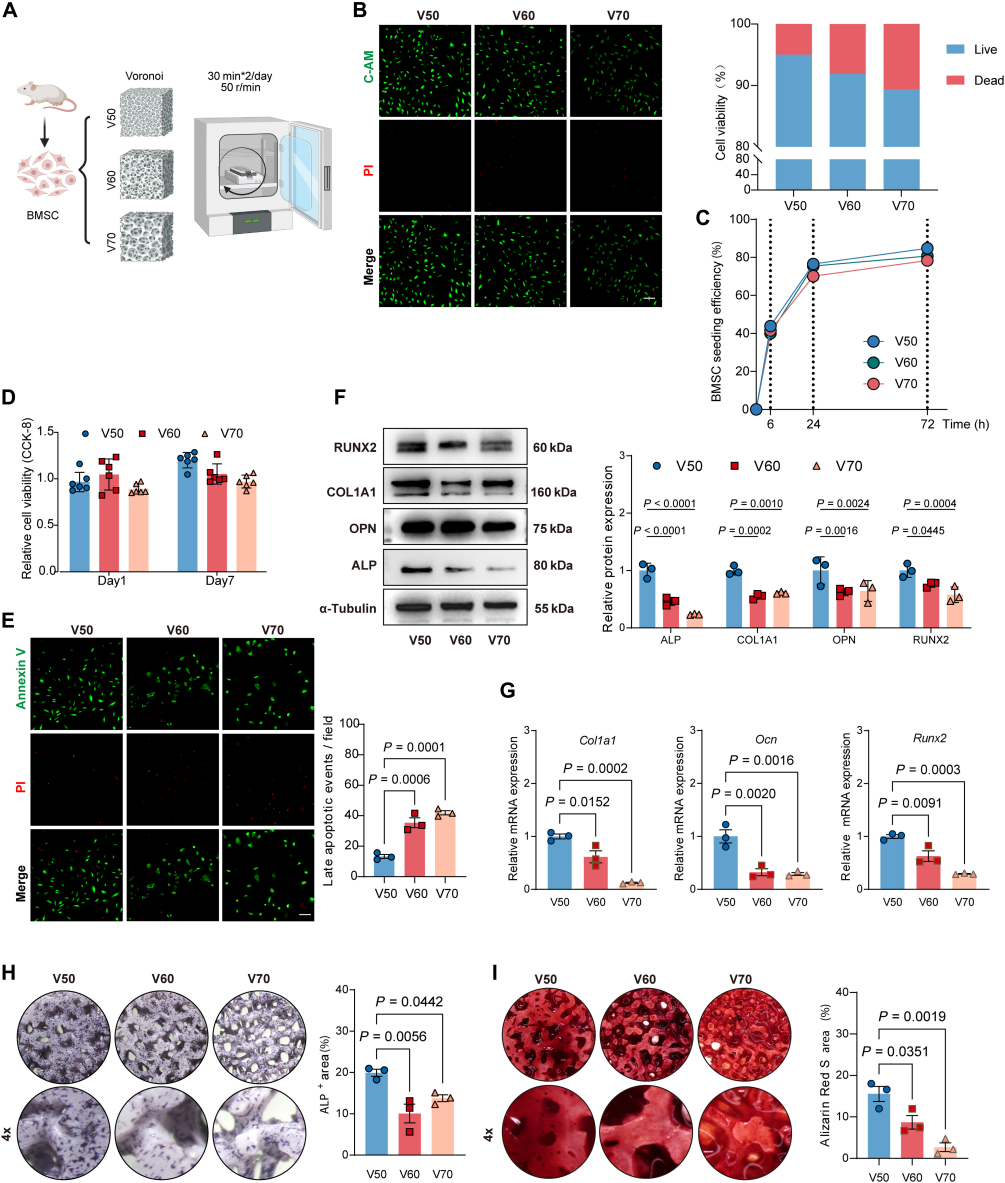
Biocompatibility and osteogenic differentiation of mouse BMSCs on Voronoi scaffolds with different porosities under FSS. (A) Schematic of the in vitro FSS culture system. Created with BioRender.com. (B) Live/dead staining of BMSCs on V50, V60, and V70 scaffolds and quantitative analysis of viable cell proportions. Scale bar, 20 μm. (C) Quantification of BMSC seeding efficiency at indicated time points. (D) CCK-8 assays of BMSC proliferation on days 1 and 7 (*n* = 6). (E) Annexin V/PI staining and quantification of apoptotic cells. Scale bar, 20 μm. (F) Western blot analysis of osteogenic proteins RUNX2, COL1A1, osteopontin, and alkaline phosphatase in BMSCs cultured on scaffolds, with quantitative analysis of protein bands normalized to α-tubulin. (G) qPCR analysis of *Runx2*, *Col1a1*, and *Ocn* mRNA expression in BMSCs on the 3 scaffolds. (H) Alkaline phosphatase staining and quantitative analysis after 7 d of osteogenic induction under FSS. (I) Alizarin Red S staining and quantification of mineral deposition after 21 d of osteogenic induction. Data are presented as mean ± standard deviation (*n* = 3 per group). Statistical significance was assessed using one-way analysis of variance with Tukey’s honestly significant difference (HSD) post hoc test.

We next asked whether, under the same dynamic stimulation framework, scaffold architecture-dependent differences in local mechanical cues further influence BMSC survival and osteogenic differentiation. Primary mouse BMSCs were seeded onto V50, V60, and V70 scaffolds and cultured on an orbital shaker at 50 rpm for 30 min twice daily in a standard incubator (Fig. 3A). Live/dead staining demonstrated good cell viability on all 3 scaffolds, while quantitative analysis revealed that the proportion of viable cells was highest on V50 and lower on V60 and V70 (Fig. 3B). To exclude the possibility that subsequent biological differences were merely caused by unequal initial cell loading, the seeding efficiency of BMSCs on V50, V60, and V70 scaffolds was evaluated at 0, 6, 24, and 72 h after seeding. No significant differences were observed among the 3 scaffold groups (Fig. 3C), indicating that comparable cell numbers were maintained on all scaffolds under the same initial seeding condition during the early culture period. Consistently, Cell Counting Kit-8 (CCK-8) assays performed on days 1 and 7 showed that BMSCs proliferated more robustly on V50 than on V60 and V70 (Fig. 3D). Annexin V/propidium iodide (PI) staining further indicated that the proportion of apoptotic cells was lowest in the V50 group, suggesting that this scaffold architecture reduces dynamic culture-associated apoptosis (Fig. 3E).

Osteogenic differentiation on the 3 scaffolds under identical dynamic culture conditions was subsequently assessed. Western blot analysis revealed that the protein expression levels of RUNX2, COL1A1, OPN, and ALP were highest in BMSCs cultured on V50, intermediate on V60, and lowest on V70 (Fig. 3F). qPCR analysis of Runx2, Col1a1, and Ocn mRNA expression reproduced this trend at the transcriptional level (Fig. 3G). Functionally, ALP staining after 7 d of osteogenic induction revealed the strongest early osteogenic response on V50, followed by V60 and V70 (Fig. 3H). After 21 d of induction, Alizarin Red S staining and quantitative analysis of mineral deposition similarly demonstrated the greatest matrix mineralization on V50 (Fig. 3I). Collectively, these results indicate that, among the 3 architectures evaluated, the V50 scaffold provides the most supportive microenvironment for BMSC survival and osteogenic differentiation under dynamic FSS.

### Transcriptomic and proteomic profiling reveals ECM remodeling and suppressed oxidative phosphorylation in BMSCs under HFSS

**Fig. 4.**
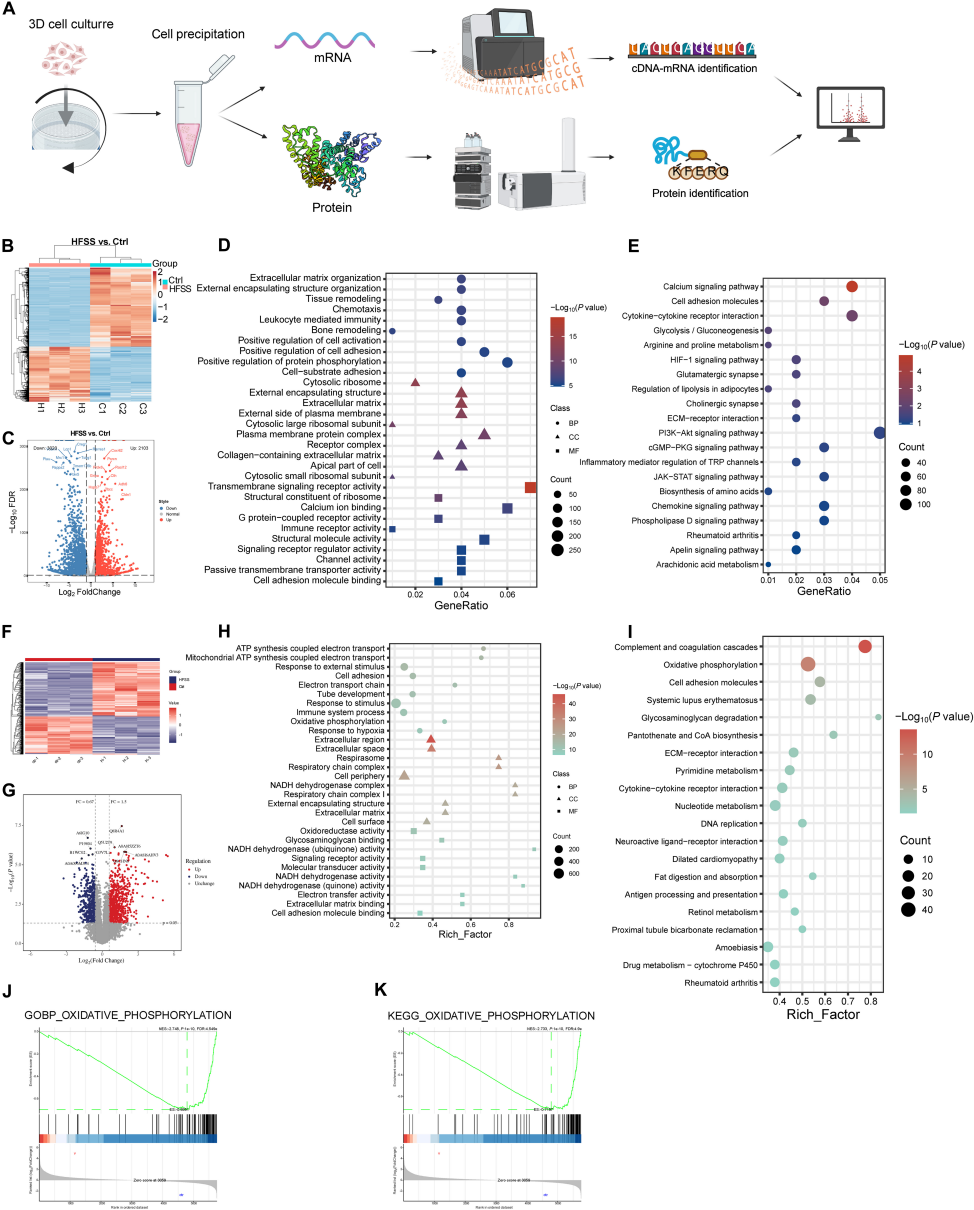
Transcriptomic and proteomic profiling of mouse BMSCs under HFSS. (A) Schematic workflow of RNA-seq and proteomic analyses. Created with BioRender.com. (B) Heatmap of DEGs between HFSS and control groups. (C) Volcano plot showing up-regulated and down-regulated genes. (D) GO enrichment analysis of DEGs in biological process, cellular component, and molecular function categories. (E) KEGG pathway enrichment analysis of DEGs. (F) Heatmap of DEPs between HFSS and control groups. (G) Volcano plot of proteins substantially altered by HFSS. (H) GO enrichment analysis of DEPs. (I) KEGG pathway enrichment analysis of DEPs. (J) GSEA plot for the GO biological process related to oxidative phosphorylation, showing negative enrichment in the HFSS group. (K) GSEA plot for the KEGG oxidative phosphorylation pathway, demonstrating substantial down-regulation under HFSS. RNA-seq and proteomic data were obtained from *n* = 3 independent samples per group. Differential expression and enrichment analyses were conducted using standard statistical criteria as described in Materials and Methods.

To obtain an unbiased understanding of how HFSS reshapes gene expression programs in BMSCs cultured on Voronoi scaffolds, parallel RNA sequencing (RNA-seq) and label-free quantitative proteomic analyses were performed on BMSCs cultured on V50 scaffolds under dynamic HFSS conditions and static control (Ctrl) conditions, followed by integrated bioinformatic analysis (Fig. 4A). At the transcriptomic level, HFSS induced extensive transcriptional reprogramming compared with Ctrl, as illustrated by the clustered heatmap of differentially expressed genes (DEGs) and the volcano plot (Fig. 4B and C). Gene Ontology (GO) enrichment analysis of DEGs highlighted biological processes and molecular functions closely associated with ECM remodeling and bone reconstruction, as well as terms related to cell adhesion and cytoskeletal organization (Fig. 4D). Consistently, Kyoto Encyclopedia of Genes and Genomes (KEGG) pathway analysis revealed substantial enrichment of cell adhesion molecules, HIF-1 signaling (Fig. S2), and PI3K–AKT signaling pathways in the HFSS group (Fig. 4E), suggesting that HFSS activates a mechanosensitive program linking adhesion-mediated sensing to osteogenic signaling.

Proteomic profiling of the same samples revealed a largely concordant yet complementary pattern at the protein level. The heatmap and volcano plot of differentially expressed proteins (DEPs) showed a clear separation between HFSS and Ctrl samples, with numerous ECM-related and metabolism-related proteins being substantially altered (Fig. 4F and G). GO enrichment analysis of DEPs again emphasized cell adhesion and ECM organization, while also highlighting oxidative phosphorylation-related terms, indicating the involvement of cellular energy metabolism under HFSS (Fig. 4H). KEGG analysis of DEPs further confirmed the enrichment of oxidative phosphorylation and cell adhesion pathways (Fig. 4I). Gene set enrichment analysis (GSEA) based on GO biological processes and KEGG pathways demonstrated negative enrichment of oxidative phosphorylation in the HFSS group (Fig. 4J and K), indicating a global down-regulation of mitochondrial respiration. Taken together, these transcriptomic and proteomic findings suggest that HFSS simultaneously promotes ECM remodeling and adhesion-related signaling while suppressing oxidative phosphorylation, implying a shift in BMSC energy metabolism that may favor glycolytic reprogramming on Voronoi scaffolds. This coordinated pattern is consistent with previous studies linking ECM remodeling and adhesion-associated mechanotransduction to metabolic reprogramming. ECM remodeling has been shown to regulate glucose metabolism, and cell–ECM mechanical signaling can reshape mitochondrial homeostasis and cellular metabolic state [29], suggesting that the HFSS-induced combination of ECM/adhesion remodeling and oxidative phosphorylation suppression observed here may reflect a coordinated mechanometabolic adaptation.

### Integrated multi-omics and in vitro validation identify a CAV1-centered reprogramming under HFSS

**Fig. 5.**
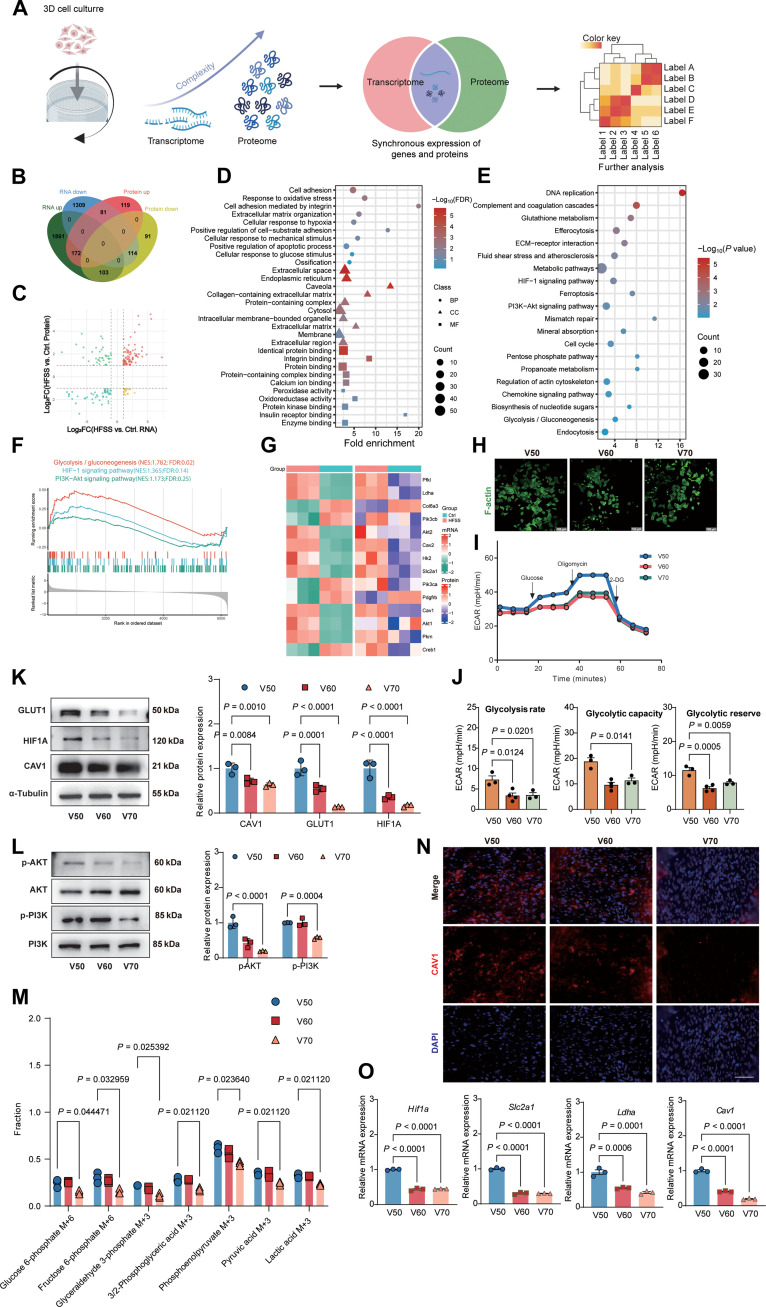
Integrated transcriptomic and proteomic analyses and in vitro validation of mechano-metabolic remodeling in BMSCs under HFSS. (A) Schematic workflow of the integrated analysis. DEGs and DEPs between the HFSS and Ctrl groups were identified from RNA-seq and proteomic datasets, respectively, and concordantly regulated targets showing the same direction of change at both transcriptomic and proteomic levels were selected for downstream analyses. Created with BioRender.com. (B) Venn diagram illustrating the overlap between DEGs and DEPs. (C) Nine-quadrant plot comparing log₂ fold changes at the RNA and protein levels, highlighting concordant and discordant regulation patterns. (D) GO enrichment analysis of concordantly regulated genes and proteins. (E) KEGG pathway enrichment analysis of concordant targets. (F) GSEA plots for glycolysis, HIF-1 signaling, and PI3K–AKT signaling, showing positive enrichment of all 3 pathways in the HFSS group. (G) Heatmap of selected genes and proteins involved in glycolysis and mechanosignaling. (H) Phalloidin staining of the actin cytoskeleton in BMSCs cultured on Voronoi scaffolds under dynamic FSS. (I) Seahorse extracellular flux analysis of BMSCs cultured on V50, V60, and V70 scaffolds. (J) Representative ECAR curves and quantitative analysis of glycolysis rate, glycolytic reserve, and glycolytic capacity. (K) Western blot analysis of GLUT1, HIF1A, and CAV1, and (L) PI3K, p-PI3K, AKT, and p-AKT in BMSCs cultured on scaffolds, with corresponding quantitative analyses of Western blot protein bands. (M) Quantitative analysis of multiple glycolytic flux fractions in BMSCs seeded onto scaffolds. (N) Representative immunofluorescence images of CAV1 expression in BMSCs cultured on V50, V60, and V70 scaffolds. Scale bar, 100 μm. (O) qPCR analysis of *Hif1a*, *Ldha*, *Slc2a1*, and *Cav1* gene expression. Data are presented as mean ± SD (*n* = 3 per group). Statistical significance was assessed using unpaired 2-tailed Welch’s *t* test and one-way ANOVA with Tukey’s HSD post hoc test.

To identify mechanical and metabolic pathways that were consistently regulated at both the transcriptomic and proteomic levels under HFSS, RNA-seq and proteomic datasets were integrated, with a focus on DEGs and proteins exhibiting concordant changes (Fig. 5A). Venn analysis revealed a substantial overlap between the 2 omics layers, with 172 genes (4.725%) or proteins (25.294%) synchronously up-regulated and 114 genes (3.132%) or proteins (16.765%) synchronously down-regulated in the HFSS group compared with the Ctrl group (Fig. 5B). A 9-quadrant plot further demonstrated that a large proportion of mechanosensing and metabolic regulators clustered in the up–up quadrant, indicating consistent induction at both the mRNA and protein levels under HFSS (Fig. 5C). GO enrichment analysis of the concordantly regulated targets highlighted biological processes including cell adhesion, response to mechanical stimulus, response to glucose, oxidative stress, and ECM organization, together with related cellular component and molecular function terms (Fig. 5D). KEGG pathway analysis of the same gene and protein set revealed substantial enrichment of metabolic pathways, HIF-1 signaling, PI3K–AKT signaling, FSS-related signaling, and glycolysis (Fig. 5E). Consistent with these results, GSEA based on glycolysis, HIF-1, and PI3K–AKT gene sets showed marked positive enrichment of all 3 pathways in the HFSS group (Fig. 5F). A heatmap of representative targets across these pathways further confirmed that HFSS robustly up-regulated both mRNA and protein levels of key genes, including *Pfkl*, *Ldha*, *Hk2*, *Slc2a1*, and *Cav1* (Fig. 5G). Collectively, the integrated analysis suggests that HFSS activates a coordinated program that links cytoskeletal and adhesion signaling with PI3K–AKT and HIF-1-driven glycolytic reprogramming. In parallel, untargeted metabolomic profiling demonstrated a clear separation between HFSS and Ctrl samples in both negative- and positive-ion modes, accompanied by extensive remodeling of metabolite profiles (Fig. S3A to F). KEGG and metabolite set enrichment analysis (MSEA) highlighted alterations in central carbon and amino acid metabolism, together with lipid- and oxidative stress-related pathways, including sphingolipid metabolism, ferroptosis, and phosphoinositide-associated signaling, further supporting HFSS-induced reorganization of energy metabolism and membrane microdomains (Fig. S3G and H).

To validate the key mechanometabolic signals identified from the integrative omics analysis, we first examined whether these candidate molecules were consistently altered under the same static and HFSS conditions used for sequencing. Immunofluorescence staining revealed increased CAV1 expression in BMSCs cultured under HFSS compared with static conditions (Fig. S4A). Consistently, Western blot analysis showed that the protein levels of CAV1, HIF-1α, GLUT1, and LDHA were markedly up-regulated under HFSS, and quantitative analysis of band intensities confirmed these changes (Fig. S4B and C). In agreement with the protein data, qPCR analysis further demonstrated that the mRNA expression levels of *Cav1*, *Hif1a*, *Slc2a1*, and *Ldha* were substantially elevated in the HFSS group (Fig. S4D).

Based on this, we next investigated whether scaffold architecture-dependent mechanical differences further modulate the magnitude of this mechanometabolic response under dynamic culture. Phalloidin staining demonstrated that BMSCs cultured on V50 scaffolds exhibited more pronounced cell spreading and well-organized actin stress fibers compared with those on V60 and V70 scaffolds, indicating stronger cytoskeletal organization on V50 under HFSS (Fig. 5H). Seahorse analysis showed that V50 supported the highest extracellular acidification rate (ECAR), with significantly increased glycolysis rate, glycolytic reserve, and glycolytic capacity relative to V60 and V70 (Fig. 5I and J), consistent with enhanced glycolytic flux. At the molecular level, Western blotting revealed that the protein levels of GLUT1, HIF1A, and CAV1 were highest in BMSCs cultured on V50 scaffolds, accompanied by marked increases in PI3K and AKT phosphorylation, indicating robust activation of the PI3K–AKT axis on this scaffold (Fig. 5K and L). In parallel, representative immunofluorescence staining showed that CAV1 expression was highest in the V50 group and lower in the V60 and V70 groups (Fig. 5N), further supporting scaffold-dependent activation of CAV1-associated mechanosignaling. The qPCR analysis further confirmed higher mRNA expression levels of the glycolytic genes *Ldha*, *Hif1a*, and *Slc2a1*, as well as *Cav1*, in the V50 group (Fig. 5O). To further validate glycolytic remodeling with a more rigorous pathway-resolved approach, we performed stable isotope-resolved metabolic tracing to directly quantify glucose-derived carbon incorporation into glycolytic intermediates in BMSCs cultured on the 3 Voronoi scaffolds. Compared with V60 and V70, BMSCs cultured on V50 scaffolds exhibited higher isotopologue fractions of multiple glycolytic metabolites (Fig. 5M), indicating enhanced glucose carbon utilization through the glycolytic pathway. Because this isotope-tracing strategy interrogates carbon flux distribution rather than relying solely on steady-state metabolite abundance, it provides high-content evidence that the lower-porosity scaffold promotes bona fide glycolytic rewiring under dynamic stimulation. Consistent with the isotopologue fraction data, supplementary analysis of labeled metabolite abundance revealed selective increases in several glycolytic intermediates, including glyceraldehyde-3-phosphate M+3 and phosphoenolpyruvate M+3, whereas other labeled metabolites did not show significant changes (Fig. S5). These results further suggest that the scaffold-dependent metabolic difference is primarily characterized by redistribution of glucose-derived carbon flux rather than a uniform expansion of all metabolite pools. Together with the Seahorse and immunoblotting results, these findings strengthen the conclusion that the V50 scaffold most effectively drives mechanometabolic adaptation in BMSCs.

To further evaluate the translational relevance of our findings, we performed a supplementary validation in human bone marrow-derived mesenchymal stem cells (hBMSCs). Flow cytometric analysis confirmed that the isolated cells exhibited the expected MSC immunophenotype (Fig. S6). When cultured on V50, V60, and V70 scaffolds, hBMSCs displayed patterns broadly consistent with those observed in mouse BMSCs: The V50 scaffold was associated with higher expression of CAV1, HIF1A, GLUT1, LDHA, and osteogenic markers, together with stronger matrix mineralization after osteogenic induction (Fig. S7A to E). These findings support that the scaffold-dependent mechanometabolic response identified in the mouse system is conserved in human BMSCs.

These data corroborate the multi-omics findings and support the conclusion that HFSS applied to the V50 Voronoi scaffold enhances cytoskeletal organization and activates a mechanical and glycolytic signaling cascade that drives metabolic adaptation in BMSCs.

### *Cav1* knockdown attenuates HFSS-induced glycolytic activation and osteogenic differentiation in BMSCs

**Fig. 6.**
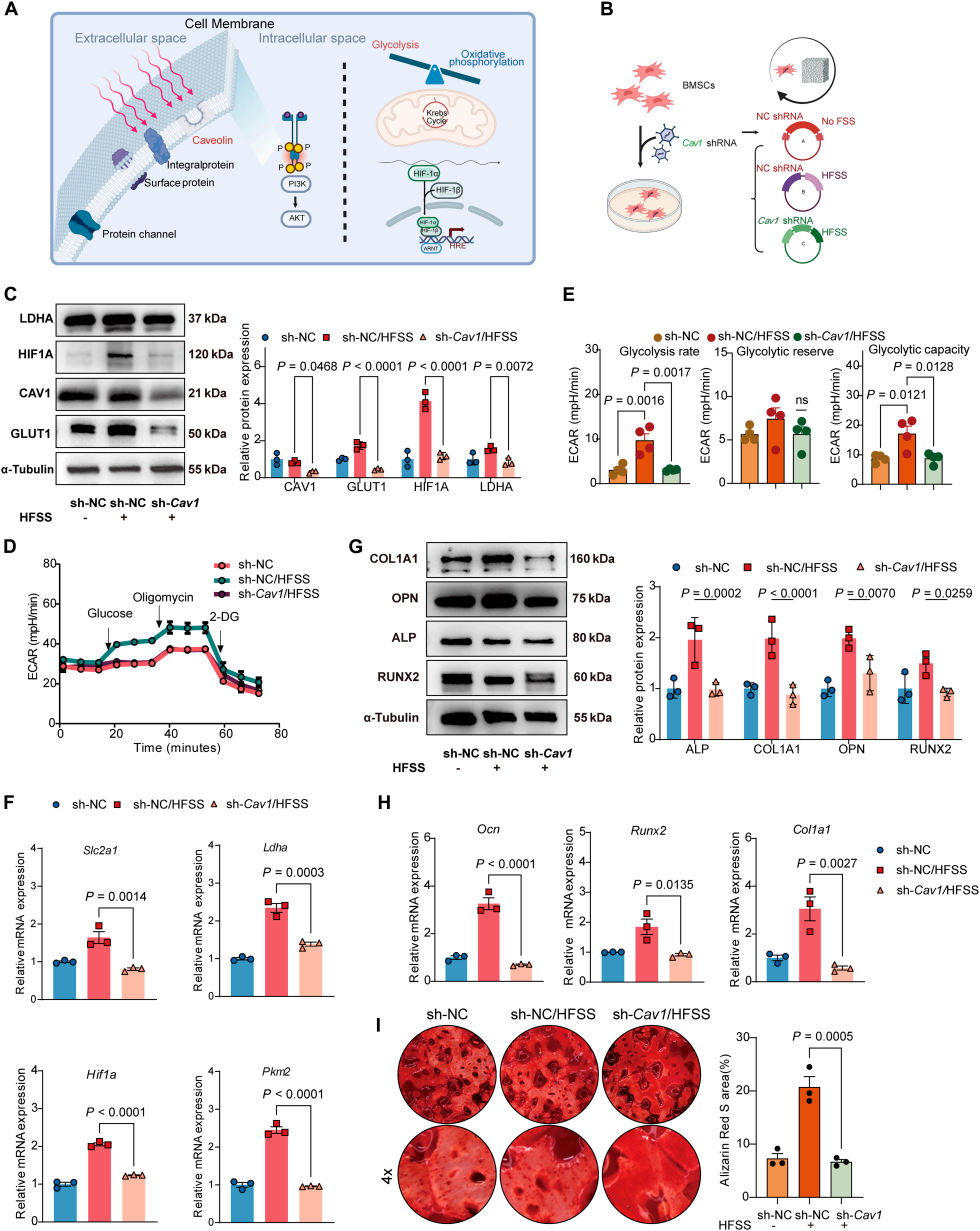
*Cav1* knockdown attenuates HFSS-induced glycolytic activation and osteogenic differentiation in mouse BMSCs. (A) Schematic illustration of the proposed CAV1-centered mechano-metabolic axis. Caveolae-associated CAV1 at the plasma membrane is hypothesized to couple mechanical cues to PI3K–AKT and HIF-1 signaling pathways, thereby promoting glycolytic metabolism. Created with BioRender.com. (B) Experimental design for *Cav1* knockdown under 3D dynamic culture conditions. BMSCs transduced with control shRNA or *Cav1* shRNA were seeded onto Voronoi scaffolds and subjected to HFSS in a 3D culture system. (C) Western blot analysis of LDHA, HIF1A, CAV1, and GLUT1 in *Cav1*-knockdown BMSCs under HFSS, with corresponding quantitative analyses of Western blot protein bands. (D) Representative ECAR curves and (E) quantitative analysis of glycolysis rate, glycolytic reserve, and glycolytic capacity. (F) qPCR analysis of *Ldha*, *Hif1a*, *Pkm2*, and *Slc2a1* messenger RNA expression in the same 3 groups. (G) Western blot analysis of osteogenic proteins COL1A1, OPN, ALP, and RUNX2 in the 3 groups. (H) qPCR analysis of *Col1a1*, *Ocn*, and *Runx2* mRNA expression. (I) Alizarin Red S staining and quantitative analysis of mineralized nodules after 21 d of 3D dynamic culture on Voronoi scaffolds. Data are presented as mean ± SD (*n* = 3 per group). Statistical significance was assessed using one-way ANOVA with Tukey’s HSD post hoc test.

Based on the multi-omics analysis, the functional role of CAV1 in linking mechanical cues to metabolic reprogramming in BMSCs was further investigated. A conceptual diagram illustrates the working model in which caveolae-associated CAV1 at the plasma membrane couples upstream mechanical stimuli to PI3K–AKT and HIF-1 signaling, thereby regulating downstream glycolysis (Fig. 6A). To directly test this hypothesis, BMSCs subjected to lentiviral knockdown of *Cav1* or transduced with a negative control short hairpin RNA (shRNA) were seeded onto Voronoi scaffolds and cultured under 3D dynamic HFSS conditions (Fig. 6B). Western blotting demonstrated that HFSS markedly increased the protein levels of LDHA, HIF1A, CAV1, and GLUT1 in control cells compared with static shRNA controls, consistent with an HFSS-induced glycolytic shift. In contrast, *Cav1* knockdown significantly attenuated these changes, with LDHA, HIF1A, and GLUT1 expression reduced toward baseline levels despite HFSS stimulation (Fig. 6C). Seahorse analysis further showed that HFSS enhanced the ECAR in control cells, whereas these HFSS-induced elevations were largely abolished in *Cav1*-knockdown BMSCs (Fig. 6D and E). The qPCR analysis of the corresponding genes *Ldha*, *Hif1a*, *Pkm2*, and *Slc2a1* revealed a similar trend at the transcriptional level (Fig. 6F). These results indicate that CAV1 is required for full activation of glycolytic metabolism in response to HFSS.

The impact of *Cav1* knockdown on HFSS-induced osteogenic differentiation was subsequently examined. Western blotting revealed that HFSS up-regulated the osteogenic proteins COL1A1, OPN, ALP, and RUNX2 in control cells, whereas this up-regulation was markedly attenuated following *Cav1* silencing (Fig. 6G). The qPCR analysis of Col1a1, Ocn, and Runx2 mRNA confirmed the same pattern, with HFSS-induced increases in osteogenic gene expression largely abolished by *Cav1* knockdown (Fig. 6H). Consistently, after 21 d of 3D dynamic culture on Voronoi scaffolds, Alizarin Red S staining and quantitative analysis showed that HFSS substantially increased calcium nodule formation in control cells, whereas mineral deposition was markedly reduced absent in *Cav1*-knockdown BMSCs under the same HFSS conditions (Fig. 6I).

To further explore whether scaffold-regulated mechanical stimulation might involve additional mechanosensitive pathways beyond the CAV1-centered axis, we re-examined the integrative omics results and identified ITGA3 as one of the few candidates showing concordant changes at both the transcriptomic and proteomic levels. Immunofluorescence analysis showed that ITGA3 expression was highest in BMSCs cultured on V50 scaffolds and lower in the V60 and V70 groups (Fig. S8A). Notably, HFSS increased ITGA3 expression, whereas *Cav1* knockdown markedly attenuated this HFSS-induced up-regulation (Fig. S8B). These findings suggest that CAV1 functions as a key upstream mediator in scaffold-regulated mechanotransduction, while integrin-related adhesion signaling, represented by ITGA3, may act as part of a broader downstream or parallel response.

Collectively, these findings demonstrate that CAV1 is indispensable for HFSS-driven glycolytic activation and osteogenic differentiation of BMSCs on Voronoi scaffolds, supporting a central role for CAV1 in the mechano-metabolic coupling identified by the multi-omics analysis.

### Pharmacological perturbation validates a CAV1-centered PI3K–HIF-1–glycolytic axis under HFSS

**Fig. 7.**
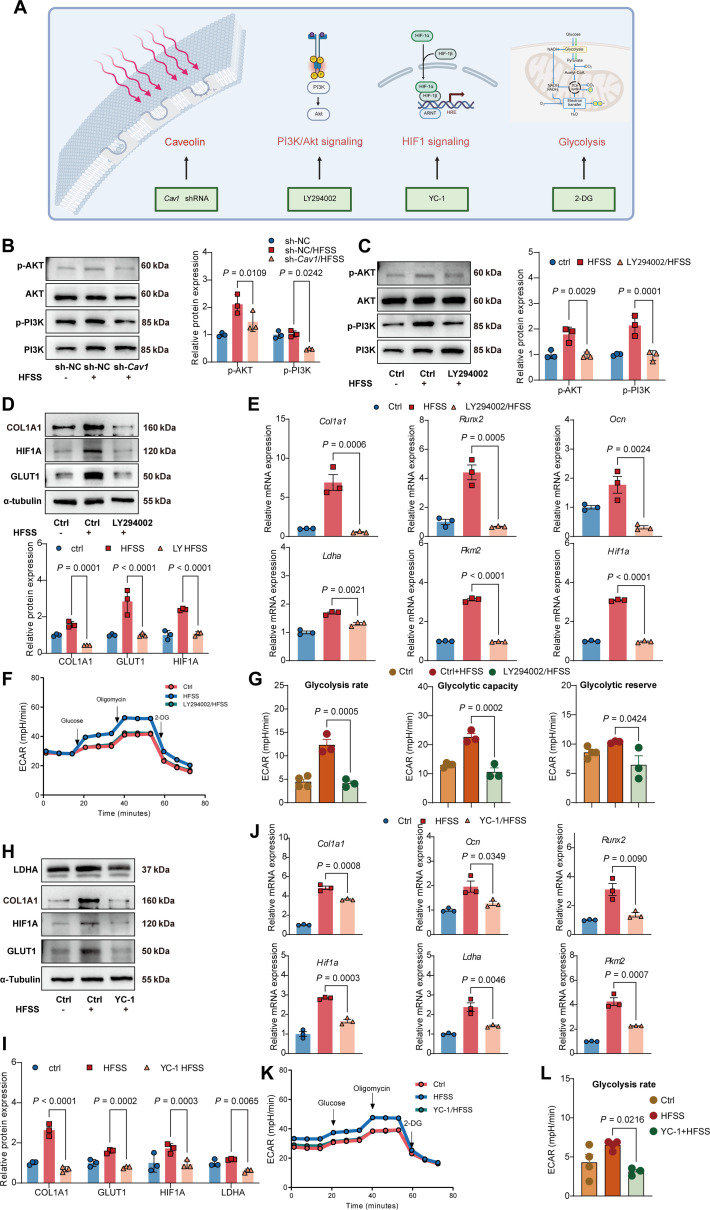
Pharmacological perturbation of the CAV1-centered mechano-metabolic axis required for HFSS-induced osteogenesis. (A) Schematic diagram illustrating the mechanistic intervention strategy. Created with BioRender.com. (B) Western blot analysis of PI3K–AKT signaling in *Cav1*-knockdown BMSCs, with corresponding quantitative analyses of Western blot protein bands. (C) Western blot analysis of PI3K, p-PI3K, AKT, and p-AKT to assess the effects of LY294002 in inhibiting the PI3K signaling pathway, with quantitative analyses of Western blot protein bands. (D) Western blot analysis of COL1A1, HIF1A, and GLUT1 expression, with corresponding quantitative analyses. (E) qPCR analysis of *Col1a1*, *Runx2*, *Opn*, *Pkm2*, *Ldha*, and *Hif1a* mRNA expression in LY294002-treated groups. (F) Representative ECAR curves and (G) quantitative analysis of glycolysis rate, glycolytic reserve, and glycolytic capacity in LY294002-treated groups. (H) Western blot analysis of LDHA, COL1A1, HIF1A, and GLUT1 to assess the effects of YC-1 in inhibiting the HIF-1 signaling pathway, and (I) quantitative analyses of Western blot protein bands. (J) qPCR analysis of *Col1a1*, *Runx2*, *Opn*, *Pkm2*, *Ldha*, and *Hif1a* mRNA expression in YC-1-treated groups. (K) Representative ECAR curves and (L) quantitative analysis of glycolysis rate in YC-1-treated groups. Data are presented as mean ± SD (*n* = 3 per group). Statistical significance was assessed using one-way ANOVA with Tukey’s HSD post hoc test.

On the basis of the *Cav1*-knockdown experiments, the downstream signaling cascade through which CAV1 couples HFSS to metabolic reprogramming and osteogenic differentiation was further investigated. A schematic diagram summarizes the experimental strategy, in which 4 key nodes along the proposed CAV1-centered PI3K–HIF-1–glycolytic axis were selectively targeted using specific inhibitors or shRNA (Fig. 7A). Western blotting confirmed that HFSS activated the PI3K–AKT pathway in negative control BMSCs cultured on Voronoi scaffolds, whereas this activation was largely abolished in the *Cav1*-knockdown group, indicating that CAV1 is required for HFSS-induced PI3K–AKT signaling (Fig. 7B).

To further interrogate the role of PI3K–AKT signaling, BMSCs were treated with LY294002 during HFSS stimulation. Compared with HFSS alone, LY294002 markedly reduced PI3K and AKT phosphorylation and concomitantly decreased the protein expression of COL1A1, HIF1A, and GLUT1 (Fig. 7C and D), indicating that PI3K–AKT activity is required for the up-regulation of both HIF-1α and glycolytic and osteogenic effectors. The qPCR analysis of osteogenic and glycolytic genes revealed a similar pattern, with HFSS-induced increases in mRNA expression significantly attenuated by LY294002 treatment (Fig. 7E). Seahorse analysis further demonstrated that HFSS enhanced the ECAR and increased the glycolysis rate, glycolytic reserve, and glycolytic capacity in vehicle-treated cells, whereas these HFSS-induced elevations in glycolytic function were largely abolished by PI3K inhibition (Fig. 7F and G). Together, these results position PI3K–AKT signaling upstream of glycolytic remodeling and osteogenesis in HFSS-stimulated BMSCs.

The contribution of HIF-1α signaling was subsequently examined using YC-1. Under HFSS conditions, YC-1 treatment reduced HIF1A protein levels and markedly decreased the expression of LDHA, COL1A1, and GLUT1 compared with HFSS alone (Fig. 7H and I), indicating that HIF-1α activity is essential for both glycolytic and osteogenic responses. Consistently, osteogenic and glycolytic mRNA levels were elevated by HFSS but were significantly suppressed in the YC-1-treated group (Fig. 7J). Seahorse analysis showed that YC-1 similarly attenuated HFSS-induced increases in ECAR and glycolysis rate (Fig. 7K and L), further supporting a critical role for HIF-1α in mediating HFSS-driven glycolytic activation. Finally, to directly determine whether glycolytic flux itself is required for the pro-osteogenic effects of HFSS, 2-deoxy-d-glucose (2-DG) was applied during dynamic culture. Western blotting revealed that 2-DG treatment under HFSS conditions markedly reduced the protein levels of COL1A1, RUNX2, and OPN compared with HFSS alone (Fig. S9A and B), which was further confirmed by mRNA expression analysis (Fig. S9C). Together with the *Cav1*-knockdown data, these findings support a model in which HFSS activates a CAV1-centered PI3K–HIF-1–glycolytic axis that is required for enhanced osteogenic differentiation of BMSCs on Voronoi scaffolds.

### In vivo bone defect repair with Voronoi-based trabecular-like scaffolds

**Fig. 8.**
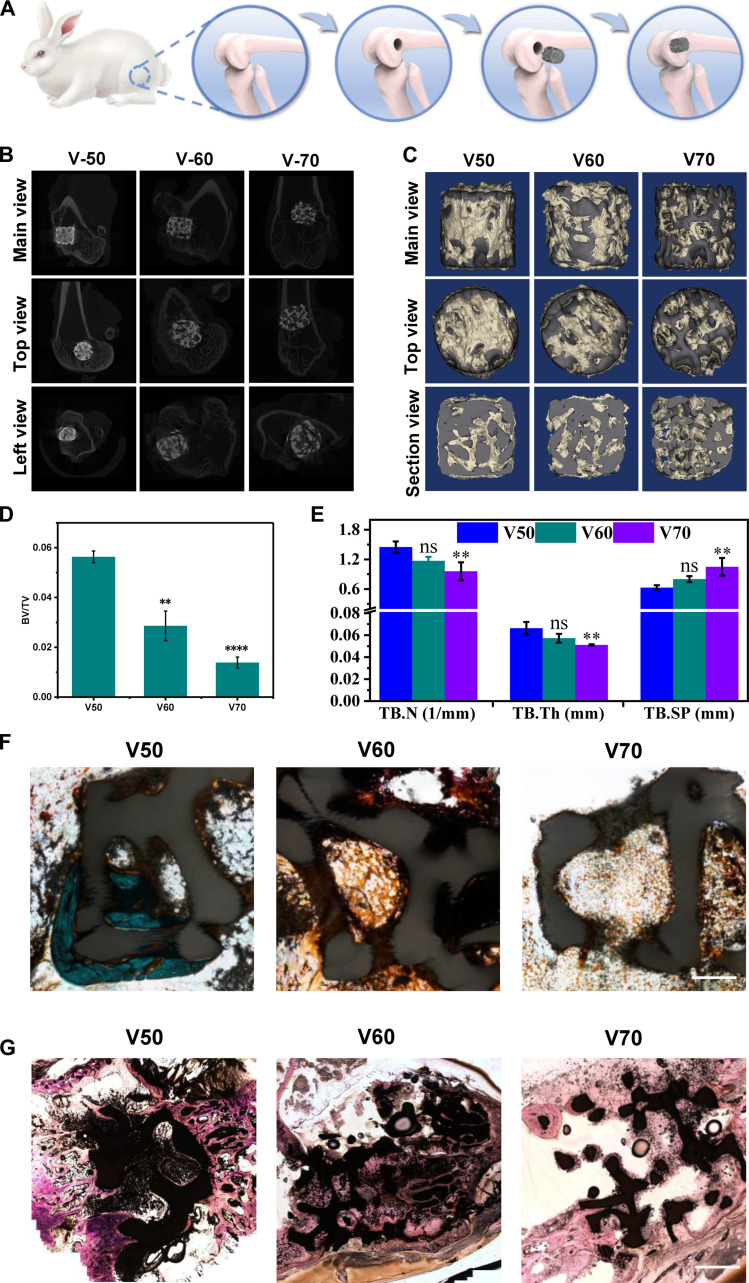
In vivo evaluation of bone regeneration in rabbit femoral defects using Voronoi-based trabecular-mimetic scaffolds. (A) Schematic diagram of the osteogenic evaluation procedure in a rabbit femoral defect model. (B) Multiplanar micro-CT reconstruction at 12 weeks post-implantation, showing axial (top), sagittal (center), and coronal (left) views of bone and scaffold integration. (C) 3D rendered micro-CT images illustrating distinct patterns of bone ingrowth among different scaffold groups after 12 weeks of implantation. Quantitative micro-CT analyses of (D) BV/TV and (E) trabecular parameters, including Tb.Th, Tb.N, and Tb.Sp. (F) Representative Goldner’s trichrome stained histological sections of the different scaffold groups. Scale bars, 100 μm. (G) Representative H&E stained histological sections of the different scaffold groups. Scale bars, 500 μm. Data are presented as mean ± SD (*n* = 6 rabbits per group). Statistical significance was assessed using one-way ANOVA with Tukey’s HSD post hoc test.

The in vivo bone regeneration performance of the bioinspired scaffolds was systematically evaluated using a rabbit femoral condyle defect model with a cylindrical defect diameter of 5 mm, as schematically illustrated in Fig. 8A. At 12 weeks post-implantation, the femoral condyles were harvested, and bone integration was initially assessed by micro-computed tomography (CT). No apparent pathological changes were observed (Fig. S10). Micro-CT images demonstrated that all 3 scaffolds remained well positioned within the distal femur without dislocation or fracture and exhibited close integration with the surrounding host bone (Fig. 8B). Newly formed bone tissue was observed coating and infiltrating the implanted scaffolds, confirming favorable osseointegration of the Voronoi-based porous architecture (Fig. 8C). Notably, the V50 scaffold promoted a thicker layer of newly formed bone compared with the higher-porosity scaffolds (V60 and V70). To further substantiate these observations, key bone morphological parameters were semi-quantified based on the micro-CT images. As shown in Fig. 8D, the bone volume fraction (BV/TV) of the V50 scaffold group reached 5.63 ± 0.23%, which was significantly higher than that of the V60 (2.86 ± 0.59%) and V70 (1.39 ± 0.22%) scaffold groups. In addition, trabecular parameters, including trabecular number (Tb.N) and trabecular thickness (Tb.Th), exhibited similar trends, whereas trabecular separation (Tb.Sp) showed an opposite pattern (Fig. 8E). These results indicate that Voronoi-based trabecular-like scaffolds with relatively lower porosity more effectively accelerate bone regeneration. Goldner trichrome staining further demonstrated well-formed and mature bone tissue within the defect region, confirming successful bone growth and healing (Fig. 8F). To further evaluate bone ingrowth at a larger spatial scale, we performed low-magnification hematoxylin and eosin (H&E) staining of the defect regions. The panoramic histological images showed more extensive newly formed bone tissue within the V50 scaffolds compared with the V60 and V70 groups (Fig. 8G). These results indicate that the V50 scaffold facilitates more effective bone ingrowth and tissue infiltration at the macroscopic level.

## Discussion

In this study, we systematically demonstrated how scaffold architecture shapes the local mechanical microenvironment to regulate stem cell metabolism and ultimately determine bone regeneration efficiency. By employing highly tunable Voronoi-inspired scaffolds, we established a causal relationship between fluid mechanical cues and intracellular metabolic programs and proposed a previously unrecognized mechano-metabolic coupling mechanism with important implications for the design of next-generation bone repair materials.

Current strategies for bone scaffold design often emphasize parameters such as porosity, pore size, and connectivity. The influence of these scaffolds on the cellular microenvironment is frequently generalized as facilitating cell migration or nutrient transport. However, pore geometry directly determines intraporous fluid mechanics, particularly the magnitude and spatial distribution of FSS [30,31]. Most existing scaffold systems are unable to vary a single geometric parameter without simultaneously altering other features, such as pore size, interconnectivity, or strut thickness. This situation closely resembles the coupled changes observed in trabecular microarchitecture during osteoporosis and has hindered efforts to decouple and rigorously investigate the dominant role of FSS.

Voronoi-based biomimetic structures overcome these limitations [32]. By mimicking the heterogeneity and interconnectivity of cancellous bone, these scaffolds allow the overall topology to remain constant, while porosity is precisely tuned through adjustment of seed-point density. This design strategy enabled the generation of scaffold models with matched geometric characteristics but distinct FSS profiles. Computational fluid dynamics (CFD) simulations confirmed substantial differences in FSS distribution among the 3 porosity groups, providing a robust engineering basis for subsequent mechanistic investigations. While recent studies on Voronoi architectures have elegantly demonstrated their phenomenological advantages such as favorable mechanical modulus, excellent fluid permeability, and macroscopic osseointegration, the intracellular molecular mechanisms linking specific fluid mechanics to stem cell fate remain largely unexplored. Our study bridges this critical gap. Rather than simply confirming osteogenic phenomena, we systemically elucidated a cross-scale mechanobiological mechanism and this shifts the paradigm of scaffold evaluation from structural phenotyping to deep metabolic regulation.

Although FSS is widely recognized as a key stimulus for osteogenic differentiation, the mechanisms by which BMSCs sense and transduce FSS into intracellular responses remain incompletely understood [33]. Multi-omics analyses revealed that the earliest and most prominent effect of FSS was not limited to activation of classical mechanotransduction pathways but involved remodeling of membrane architecture, most notably through pronounced up-regulation of CAV1. As a mechanical stabilizer of the plasma membrane, CAV1 responds to increased membrane tension and contributes to the maintenance of membrane integrity under elevated FSS [34]. The present findings indicate that CAV1 up-regulation represents both an adaptive response to altered membrane stress and a structural platform that reorganizes downstream signaling.

Previous studies have shown that CAV1 activity can potentiate PI3K–AKT signaling [35]. The *Cav1* knockdown and PI3K inhibition experiments performed in this study further verified this causal relationship, identifying CAV1 as a molecular bridge that amplifies AKT signaling in response to FSS. AKT signaling is a well-established stabilizer of HIF-1α, a master regulator of glycolytic reprogramming [36–38]. Consistent with this mechanism, BMSCs cultured on HFSS scaffolds exhibited robust activation of HIF-1α and enhanced glycolytic activity. Rapid ATP generation and anabolic precursor production are critical during the early stages of osteogenic differentiation, and glycolysis provides these resources efficiently. Inhibition of glycolysis using 2-DG or suppression of HIF-1α using YC-1 markedly reduced osteogenic gene expression, demonstrating that metabolic reprogramming is indispensable for FSS-driven osteogenesis.

Although mechanical stimulation and glycolysis have each been independently linked to osteogenesis, few studies have integrated these factors into a unified pathway [39,40]. Here, we propose a cross-scale regulatory mechanism in which low-porosity trabecular scaffolds generate higher FSS, thereby activating a CAV1-mediated PI3K–AKT/HIF-1α axis that drives glycolytic reprogramming. This metabolic shift provides the energy supply and biosynthetic support required for osteogenic differentiation of BMSCs. These results explain why HFSS scaffolds more effectively promote bone regeneration in vivo and highlight how material architecture regulates cellular metabolism through a bone-specific mechanical microenvironment.

It is crucial to emphasize that the in vivo translation of this FSS-driven mechanism is facilitated by the inherently dynamic nature of the physiological bone microenvironment. Rather than being a static void, the loaded bone tissue drives interstitial fluid flow through the marrow cavity. To evaluate this, the structural parameters were adjusted to generate 3 distinct scaffold groups with macro-porosities of 55%, 65%, and 75%. This specific porosity range was rationally selected to biomimetically match the physiological properties of natural human cancellous bone, which typically exhibits a porosity of 50% to 90% and an elastic modulus of 0.1 to 2 GPa. Porosities lower than 55% were deliberately excluded from the study design. From a biomechanical standpoint, excessively low porosities yield an elastic modulus approaching that of cortical bone, risking severe stress shielding upon implantation. Furthermore, biologically, maintaining a porosity above the 55% threshold ensures adequate fluid permeability, which is strictly required for interstitial flow-mediated mechanotransduction, efficient nutrient/waste exchange, and the prevention of core cell necrosis during deep bone ingrowth. Our CFD simulation applied a dynamic inlet velocity of 10 mm/s. Results demonstrated that the specific microarchitecture of the V50 scaffold locally accelerates this macroscopic flow to generate a maximum FSS of 1.14 Pa, which falls perfectly within the widely recognized optimal physiological window (0.5 to 3.0 Pa) for promoting osteogenic differentiation [41,42]. In contrast, V60 and V70 scaffolds only reached 0.65 and 0.53 Pa, respectively, providing weaker mechanical stimulation. Therefore, upon in vivo implantation, the low-porosity scaffold acts as an effective physical modulator, translating natural macroscopic physiological flow into osteoinductive microlevel FSS in situ to activate the CAV1-mediated metabolic pathways.

Furthermore, according to the fundamental principles of laminar fluid dynamics within defined porous geometries, the FSS (τ) is linearly proportional to the macroscopic fluid velocity (v). This linear relationship (τ∝v) imparts marked physiological robustness to our findings. In vivo, the actual interstitial fluid velocity within the bone marrow cavity dynamically fluctuates depending on the host's physical activity levels. However, because of this linear proportionality, the relative mechanical advantage of the V50 scaffold remains invariant. Regardless of whether the physiological flow is slow during rest or fast during exercise, the V50 architecture consistently serves as a structural amplifier, generating a higher and more optimal osteoinductive FSS magnitude than its higher-porosity counterparts. This intrinsic geometric property ensures sustained mechanical stimulation under complex and variable in vivo physiological environments.

While perfusion bioreactors are commonly used to apply steady unidirectional flow, we employed an orbital shaking system to provide dynamic mechanical stimulation. The orbital shaking model is highly advantageous for large-scale multi-omics sampling as it ensures high-throughput, parallel biological replication while avoiding the batch effects common in complex perfusion systems. More importantly from a physiological perspective, the multidirectional oscillatory FSS generated by orbital shaking closely mimics the complex, non-unidirectional interstitial fluid movement experienced by trabecular bone during dynamic daily activities.

Despite these advances, several limitations of the present study should be acknowledged. Although animal experiments confirmed the regenerative potential of the scaffolds, whether the actual in vivo distribution of FSS fully corresponds to the simulated predictions requires further verification. Moreover, because zirconia was used for 3D printing of the Voronoi scaffolds, hard tissue sectioning was required for animal samples, which precluded direct detection of changes in CAV1 and its downstream signaling molecules in vivo. Future studies could attempt to extract proteins or RNA from the tissue–scaffold interface immediately after harvesting to provide in vivo validation of the proposed signaling axis. In addition, beyond the CAV1/HIF-1 signaling axis, FSS may also regulate other metabolism-related pathways, including adenosine monophosphate-activated protein kinase (AMPK), mammalian target of rapamycin (mTOR), and YAP/TAZ signaling [43,44]. Notably, CAV1 has been implicated in the regulation of YAP/TAZ activity through modulation of cytoskeletal tension and membrane mechanics [45]. It would therefore be valuable to investigate whether CAV1 serves as a common upstream regulator of both HIF-1α and YAP/TAZ pathways in response to FSS, or whether these pathways operate in parallel and synergistically. Our integrative omics analysis identified ITGA3 as a consistently altered mechanosensitive candidate. Its HFSS-induced up-regulation was reduced upon CAV1 knockdown, indicating partial regulation by CAV1. This supports a role for CAV1 as an upstream mediator within a broader adhesion-related mechanotransduction network. Further in-depth investigation of these mechanisms will contribute to a more comprehensive understanding of how the mechanical environment influences stem cell metabolic fate determination. Subsequent studies may also examine the differential responses of distinct cell subsets and explore the development of adaptive materials capable of dynamically regulating mechanical cues during tissue repair.

In vivo bone regeneration is inherently multifactorial. Accordingly, we interpret the in vivo outcomes as an integrated advantage of biomimetic porosity design rather than a direct readout of a single FSS variable. Importantly, our controlled in vitro experiments establish a causal mechanistic framework. By constructing scaffolds based on a Voronoi architecture, this study achieved precise regulation of the FSS microenvironment and systematically demonstrated that mechanical stimulation drives glycolytic reprogramming and promotes osteogenic differentiation through activation of a CAV1-dependent mechanosensory mechanism. These findings establish a critical link between mechanical signals and stem cell metabolism and provide a theoretical basis for the rational design of bone tissue engineering scaffolds that integrate structural optimization with functional compatibility.

## Conclusion

**Fig. 9.**
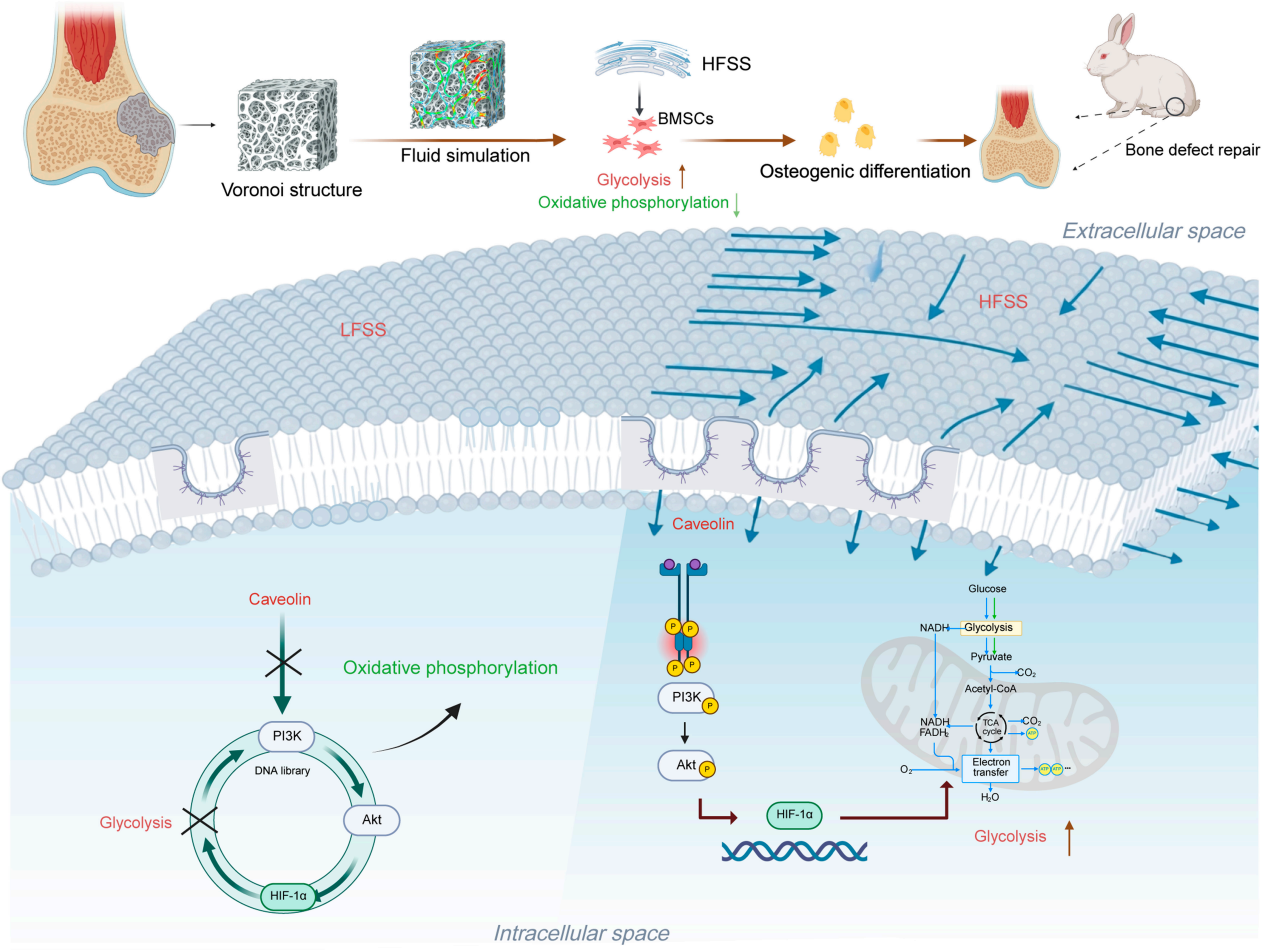
Schematic illustration of the CAV1-centered mechano-metabolic axis by which Voronoi scaffold-regulated FSS drives glycolytic reprogramming and osteogenic differentiation of BMSCs. Created with BioRender.com.

In summary, the present study substantially advances the understanding of how scaffold architecture regulates osteogenic outcomes by shaping the local fluid mechanical microenvironment and cellular metabolism. We demonstrate that FSS generated by Voronoi-based trabecular-like scaffolds activates a CAV1-dependent PI3K–AKT/HIF-1α signaling pathway, thereby driving glycolytic reprogramming to support osteogenic differentiation (Fig. 9). This work underscores the central role of CAV1-mediated mechanosensing in linking architectural design to metabolic adaptation during bone regeneration and provides new mechanistic insight into fluid-driven osteogenesis. Collectively, these findings identify scaffold geometry as an active regulator of cell metabolism and suggest promising strategies for the design of mechanically instructive biomaterials to enhance bone repair.

## Materials and Methods

### Design, materials, and fabrication

The trabecular-like porous structures were designed based on a Voronoi diagram with probability balls, an irregular biomimetic structural design method proposed in our previous studies [20,46]. Scaffolds with different porosities (V50 = 55%, V60 = 65%, and V70 = 75%) were modeled using the Grasshopper plugin embedded in the computer-aided design software Rhinoceros (Robert McNeel, USA). All samples were fabricated using a material jetting-based 3D printing process with an inert bioceramic zirconia material, employing a commercial XJET Carmel 1400 system (Rehovot, Israel) with zirconia ink containing 45 wt % ceramic particles and a support material composed of 31 wt % sodium carbonate. Following printing, all green bodies underwent debinding to remove organic binders, followed by sintering to achieve full densification. ESEM and EDS mapping (Quattro S, Thermo Fisher Scientific, USA) were performed to characterize surface morphology and elemental composition of the printed scaffolds.

### Mechanical characteristics and fluid dynamics characterization

Quasi-static compression tests were performed using an electronic universal testing machine (UTM5105, SUNS, China) equipped with a 100-kN load cell. According to GB/T 1964-2023, cubic specimens (*L* = 12 mm, *n* = 10) were compressed at a loading rate of 1.5 mm/min. To further elucidate fracture behavior, quasi-static axial compression simulations based on finite element analysis (FEA) were conducted using the commercial software ABAQUS (Dassault Systèmes Simulia, USA) with the nonlinear geometry option enabled. The 3D scaffold models were meshed using C3D10 elements. A brittle constitutive Johnson–Holmquist II (JH-2) model with standard zirconia material parameters was applied [47]. CFD analysis of the trabecular-like porous structures was performed using COMSOL Multiphysics software (COMSOL, Sweden) with single-phase peristaltic flow models. The computational fluid domain was generated by Boolean subtraction of the cubic scaffold (*L* = 6 mm) from a surrounding cube (6 × 6 × 7 mm^3^) and subsequently volume meshed using the STL editing software 3-matic (Materialise, Belgium). Two opposite faces of the cubic porous fluid domain were defined as the inlet and outlet boundaries, with an inlet velocity of 10 mm/s applied at the inflow face. This inlet velocity represents a standardized flow condition used to computationally compare the intrinsic hydraulic properties and FSS-generating potential, rather than a direct replication of the dynamic culture environment. The remaining lateral faces were defined as symmetry boundaries, and the internal scaffold surfaces were defined as no-slip walls. Fluid flow was approximated using a single-phase stationary laminar flow model. The fluid properties were assigned to simulate Dulbecco’s modified Eagle’s medium (DMEM), with a dynamic viscosity *μd* of 1.45 mPa·s and a density of 1 g/cm^3^. The computed parameters included the average pressure drop (*ΔP*), Darcy velocity (*v*_D_, defined as the superficial outlet velocity), and surface shear rate (*γ*). The permeability (*K*) of the scaffolds was calculated according to Darcy’s law, as shown in Eq. (3), and the surface shear stress (*τ*) was calculated according to Eq. (4).

$$K=\nu_{D}\mu_{d}\cdot \left(\frac{L}{\Delta P}\right)$$ (3)

$$\tau =\mu_{d}\cdot \gamma$$ (4)

### In vivo study

Male New Zealand rabbits weighing approximately 3.5 kg were obtained from the Laboratory Animal Center of Drum Tower Hospital, Medical School of Nanjing University, China. All surgical procedures were performed under aseptic conditions, and rabbits were anesthetized using lidocaine and propofol.

The osteogenic capacity of the Voronoi-based trabecular-like scaffolds was systematically evaluated using a rabbit femoral condyle defect model with a cylindrical defect of 5 mm in diameter. This defect size is established as a critical-sized defect (CSD) in rabbits, as spontaneous healing does not occur over the animal’s lifetime, thereby providing a rigorous model for assessing the bone regenerative potential of biomaterials. Cylindrical defects were created using a trephine drill and immediately implanted with the scaffolds. Postoperative infection prophylaxis was administered by intramuscular injection of penicillin for 3 d. All animals were housed under standardized conditions with daily monitoring and were euthanized at 3 months post-implantation. Harvested specimens were collected for subsequent analyses.

Retrieved bone specimens were scanned using a VivaCT-80 micro-CT system (Scanco Medical, Switzerland). Bone morphometric parameters, including BV/TV, Tb.N, Tb.Th, and Tb.Sp, were quantified based on the micro-CT data. 3D reconstructions were generated using MIMICS 19.0 software (Materialise, Belgium) with a uniform grayscale threshold, and cross-sectional analyses were performed using Magics 19.01 (Materialise, Belgium). For histological evaluation, specimens were fixed in 4% neutral buffered formalin at 4 °C for 24 to 48 h, rinsed, dehydrated through a graded ethanol series, and embedded in polymethyl methacrylate (PMMA) without decalcification. Sections with a thickness of 30 μm were prepared along the longitudinal axis using a diamond microtome (310 CP, EXAKT, Germany) and stained with Goldner trichrome. Stained sections were examined under brightfield and fluorescence microscopy (BX53, Olympus, Japan).

Major organs were carefully harvested, rinsed with saline to remove residual blood, and fixed in 4% paraformaldehyde for at least 24 h. Tissues were subsequently dehydrated through a graded ethanol series, cleared in xylene, and embedded in paraffin. Paraffin blocks were sectioned at a thickness of 5 μm, mounted on glass slides, and stained with H&E following standard protocols. Histological morphology was examined under a light microscope, and representative images were acquired to evaluate potential tissue toxicity or pathological alterations associated with scaffold implantation. Serum levels of routine biochemical parameters were measured using an automatic biochemical analyzer according to the manufacturer’s instructions.

### Cell culture of primary BMSCs

Primary mouse bone marrow mesenchymal stem cells (BMSCs) were purchased from Wuhan Procell Biotechnology Co. Ltd. and cultured in DMEM/F12 supplemented with 10% fetal bovine serum (ExCell Bio) and 1% penicillin–streptomycin. 

Primary human BMSCs (hBMSCs) were isolated from bone marrow samples obtained from donors undergoing orthopedic procedures at the Affiliated Drum Tower Hospital of Nanjing University Medical School. All procedures were approved by the Ethics Committee and written informed consent was obtained from all participants.

Mononuclear cells were isolated by Ficoll density gradient centrifugation and cultured in DMEM/F12 supplemented with 10% fetal bovine serum (fetal bovine serum, advanced, F102-01, Vazyme Biotech Co. Ltd.) and 1% penicillin–streptomycin. Non-adherent cells were removed after 48 h, and the medium was changed every 2 to 3 d. Cells were passaged at 80% to 90% confluence, and passages 3 to 5 were used for experiments.

For phenotypic characterization, hBMSCs were analyzed by flow cytometry. Cells were collected, washed, and incubated with fluorophore-conjugated antibodies against CD73 [phycoerythrin (PE)], CD90 (PE), human leukocyte antigen (HLA)-DR (PE), CD14 [fluorescein isothiocyanate (FITC)], and CD19 (FITC) (BD Pharmingen), and CD105 (PE), CD44 (PE), CD45 (FITC), CD34 (FITC), and HLA-DQ (FITC) (Thermo Fisher Scientific) according to the manufacturers’ instructions. Appropriate isotype controls were included to define positive and negative populations. Debris and doublets were excluded by sequential gating, and single-cell populations were analyzed. The isolated cells showed positive expression of CD44, CD73, CD90, and CD105, and were negative for CD14, CD19, CD34, CD45, HLA-DR, and HLA-DQ, consistent with the minimal phenotypic criteria for mesenchymal stem cells.

Voronoi-based scaffolds with porosities of 55%, 65%, and 75% (V50, V60, and V70, respectively) were placed in standard multi-well plates and seeded with BMSCs at a defined density. Following an initial attachment period under static conditions, the plates were transferred to an orbital shaker within a CO₂ incubator. Dynamic culture was performed at 50 rpm for 30 min twice daily, with cells maintained in complete medium or osteogenic medium between stimulation periods. Unless otherwise specified, exposure to HFSS was continued for the indicated durations. Cell proliferation was assessed with a commercially available CCK-8 kit (B46790, Innochem) following the manufacturer’s protocols. To ensure that downstream biological readouts were strictly reflective of FSS-induced changes rather than confounded by potential differences in cell attachment area, all quantitative molecular data were rigorously normalized to endogenous reference genes or total intracellular protein concentrations to assess the per-cell osteogenic and metabolic responses.

### Osteogenic induction and pharmacological treatments

For osteogenic induction, mBMSCs seeded on Voronoi-based scaffolds were cultured in osteogenic medium (MUXMX-90021) obtained from Cyagen Biosciences (Suzhou, China). The hBMSCs were treated with osteogenic induced differentiation medium for human bone marrow mesenchymal stem cell (D3504, Beijing Solarbio Science & Technology Co. Ltd.). The osteogenic medium was refreshed every 2 to 3 d. Pharmacological inhibitors or corresponding vehicle controls were added shortly before the initiation of HFSS stimulation and maintained throughout the stimulation period. All small-molecule compounds were purchased from Med Chem Express (Shanghai, China), including LY294002 (1 μM, catalog no. HY-10108), YC-1 (1 μM, catalog no. HY-14927), and 2-DG (1 mM, catalog no. HY-13966).

### ALP and Alizarin Red S staining and quantification

For alkaline phosphatase (ALP) staining, BMSCs cultured on scaffolds were fixed after 7 d of osteogenic induction and stained using a commercial ALP staining kit according to the manufacturer’s instructions. For assessment of late-stage mineralization, cells were fixed after 21 d of osteogenic induction and stained with 1% Alizarin Red S solution. ALP and Alizarin Red S staining were quantified using ImageJ as the percentage of positive area relative to the scaffold area within the corresponding field of view.

### Seahorse XF glycolysis stress test

Glycolytic function was evaluated using a Seahorse XF analyzer according to the manufacturer’s protocol. BMSCs subjected to 3D culture and mechanical stimulation were seeded into XF cell culture microplates at an optimized density. On the day of analysis, cells were equilibrated in Seahorse assay medium without glucose and then subjected to a glycolysis stress test with sequential injections of glucose, oligomycin, and 2-DG. ECAR was recorded in real time. Glycolysis rate, glycolytic capacity, and glycolytic reserve were calculated based on ECAR changes as defined by the manufacturer and normalized to cell number. For Seahorse assays, *n* = 3 to 4 valid technical replicate wells per group because of plate-layout constraints, and all valid wells were retained for analysis.

### Integrated analysis of multi-omics data

To investigate the molecular responses to HFSS, BMSCs cultured on V50 scaffolds were subjected to either dynamic culture (HFSS group) or static culture (control group). After culture, cells within the scaffolds (approximately 1 × 10^7^ cells per sample) were lysed for multi-omics analyses. Transcriptomic, proteomic, and untargeted metabolomic analyses were performed by Hipro Life Sciences Co., Ltd. (China). DEGs identified by RNA-seq and DEPs identified by label-free quantitative proteomics were screened between the HFSS and control groups using predefined thresholds of fold change (|log₂FC| > 0.5) and adjusted *P* value (<0.05). Gene symbols were used to match transcripts and proteins, and concordantly regulated targets were defined as DEGs and DEPs exhibiting the same direction of regulation. Overlaps between DEGs and DEPs were visualized using UpSet plots, and RNA–protein log₂ fold changes were compared using 9-quadrant plots. GO and KEGG pathway enrichment analyses were conducted for DEGs, DEPs, and concordant targets using standard enrichment tools with multiple-testing correction. GSEA was performed for selected pathways. Protein–protein interaction networks were constructed for selected modules using the STRING database and visualized with Cytoscape. RNA-seq and proteomic analyses were performed using 3 biological replicates per group (*n* = 3).

For metabolomic analysis, cell lysates were prepared and extracted metabolites were analyzed by liquid chromatography coupled with tandem mass spectrometry (LC-MS/MS) using a system equipped with an ACQUITY UPLC BEH Amide column. Gradient elution was performed using mobile phases consisting of water containing ammonium formate and formic acid and acetonitrile–water containing ammonium formate and formic acid. The mass spectrometer was operated in both positive and negative ion modes with a full scan range of 70 to 1,000 m/z. Raw data were processed and normalized using XCMS software, followed by peak identification and quantification. Differential metabolites were identified based on a fold-change threshold (|log₂FC| > 1) and a *P* value of <0.05. Pathway enrichment analysis was conducted using KEGG and MetaboAnalyst to identify substantially enriched metabolic pathways.

### Western blotting

For Western blotting, cells or scaffold–cell constructs were lysed in ice-cold radioimmunoprecipitation assay buffer supplemented with protease and phosphatase inhibitors (KeygenBioTECH). Protein concentrations were determined using a bicinchoninic acid assay (AKPR017, Beijing Boxbio Science & Technology Co. Ltd.). Equal amounts of protein were separated by sodium dodecyl sulfate–polyacrylamide gel electrophoresis and transferred onto polyvinylidene fluoride membranes. Membranes were blocked with ready-to-use rapid blocking solution (Guangzhou Biolight Biotechnology Co. Ltd.) at room temperature and incubated overnight at 4 °C with primary antibodies against COL1A1 (ab255809, Abcam), RUNX2 (ab236639, Abcam), OPN (49995, Signalway Antibody), ALP (A0514, ABclonal), CAV1 (3267, Cell Signaling Technology), HIF1A (A26889, ABclonal), GLUT1 (12939, Cell Signaling Technology), LDHA (3582, Cell Signaling Technology), p-AKT (4060, Cell Signaling Technology), AKT (4691, Cell Signaling Technology), p-PI3K (17366, Cell Signaling Technology), PI3K (4257, Cell Signaling Technology), and α-tubulin (A6830, ABclonal). Membranes were then incubated with appropriate horseradish peroxidase-conjugated secondary antibodies. Protein bands were visualized using enhanced chemiluminescence and imaged with a digital imaging system. Band intensities were quantified using ImageJ software and normalized to α-tubulin. Quantification of the PI3K–AKT signaling pathway was performed by normalizing phosphorylated proteins to their corresponding total protein levels.

### Quantitative real-time PCR

Total RNA was extracted from BMSCs or scaffold–cell constructs using TRIzol reagent or a column-based extraction kit (RNApure Fast Tissue & Cell Kit, CW0599S, CWBIO, China) according to the manufacturer’s instructions. One microgram of RNA was reverse-transcribed into complementary DNA (cDNA) using a commercial reverse transcription kit. Quantitative real-time PCR (qRT-PCR) was performed using SYBR Green chemistry on a real-time PCR system. Relative mRNA expression levels were calculated using the 2^−ΔΔCt^ method. Primer sequences used in this study are listed in Table S1.

### Lentiviral shRNA knockdown of *Cav1*

BMSCs with stable *Cav1* knockdown were generated using lentiviral shRNA (GCTTCCTGATTGAGATTCA) obtained from Hanbio Biotechnology Co., Ltd. (Shanghai, China). Briefly, BMSCs at 50 to 70% confluence were infected with lentiviral particles encoding shRNA targeting *Cav1* or a nontargeting control shRNA in the presence of polybrene. After 48 h, the culture medium was replaced with fresh complete medium, and cells were allowed to recover prior to selection with puromycin for several days until non-infected cells were eliminated. The efficiency of *Cav1* knockdown was confirmed at both the mRNA and protein levels by qRT-PCR and Western blotting before subsequent experiments.

### Fluorescence staining

For live and dead staining, BMSCs cultured on Voronoi-based scaffolds were incubated with a commercial live and dead viability kit (KTA1001, Abbkine) containing calcein acetoxymethyl ester and PI, according to the manufacturer’s instructions. After staining, scaffolds were gently rinsed with phosphate-buffered saline (PBS) and imaged using fluorescence microscopy. Live and dead cells were quantified from multiple randomly selected fields using ImageJ software.

For apoptosis analysis, cells on scaffolds were incubated with fluorophore-conjugated Annexin V and PI following the manufacturer’s protocol (BA00101, Bioss USA). Samples were washed with binding buffer and imaged under a fluorescence microscope. The proportions of Annexin V-positive and PI-negative cells representing early apoptosis and Annexin V-positive and PI-positive cells representing late apoptosis or necrosis were quantified.

For cytoskeleton staining, BMSCs cultured on Voronoi-based scaffolds were fixed with 4% paraformaldehyde, permeabilized with 0.1% Triton X-100, and blocked with 1% bovine serum albumin. Filamentous actin was labeled using fluorescent phalloidin (16001, zenbio), and nuclei were counterstained with 4′,6-diamidino-2-phenylindole (DAPI). After washing, actin cytoskeleton organization and nuclear morphology were visualized using confocal or fluorescence microscopy. Cell spreading area and stress fiber organization were analyzed using ImageJ or equivalent image analysis software.

For immunofluorescence staining of CAV1 and ITGA3 in scaffold-cultured cells, BMSCs cultured on scaffolds were fixed with 4% paraformaldehyde, permeabilized with 0.1% Triton X-100, and blocked with 1% bovine serum albumin (BSA). Samples were then incubated overnight at 4 °C with primary antibodies against CAV1 or ITGA3 (ABclonal, China), followed by incubation with the corresponding fluorescent secondary antibodies. After washing with PBS, cell nuclei were counterstained with DAPI. Fluorescence images were acquired using an Olympus upright fluorescence microscope. Fluorescence intensity was quantified using ImageJ software.

### Stable isotope-resolved glucose metabolic flux analysis

Before metabolic flux analysis, cells were preconditioned by replacing the culture medium with complete DMEM containing 3 mM glucose and incubated for 24 h. On the day of the experiment, cells were washed and incubated in glucose-free DMEM for 30 min to deplete intracellular glucose pools. The medium was then replaced with DMEM containing 10 mM ^13^C_6_-glucose, and cells were incubated at 37 °C for 30 min. Each condition was analyzed in triplicate. To assess glycolytic flux, intracellular metabolites were extracted using 80% methanol. Extracted metabolites were analyzed by liquid chromatography–high-resolution mass spectrometry (LC–HRMS; Thermo Vanquish Flex coupled with QE-HFX) using a targeted selected ion monitoring (tSIM) acquisition method. Chromatographic separation was performed on a BEH Amide column with acetonitrile and ammonium acetate-based aqueous buffers. Mass isotopomer distributions were determined for glycolytic and related metabolites, and isotopologue fractions were calculated after natural isotope correction using Skyline and MAVEN. The incorporation of ^13^C-labeled glucose into downstream metabolites was used to evaluate pathway-specific carbon flux rather than steady-state metabolite abundance. Hangzhou Cosmos Wisdom Biotech Co. Ltd. carried out glucose metabolic flux measurements, and Y. Qin assisted with the related data analysis.

### Statistical analysis

Statistical analyses were performed using SPSS 22.0 software (IBM Corp., Armonk, NY, USA). Pairwise comparisons between groups were conducted using independent-samples *t* tests or unpaired 2-tailed Welch’s *t* test. Data obtained from 3 or more independent experiments were analyzed using one-way analysis of variance (ANOVA) followed by Tukey’s honestly significant difference post hoc test and are presented as mean ± standard deviation (SD). Differences with *P* values of <0.05 were considered statistically significant at a 95% confidence interval.

## Ethical Approval

All animal experiments were approved by the Institutional Animal Care and Use Committee of Drum Tower Hospital and conducted in accordance with relevant guidelines (approval no.: 2024AE06002). Human bone marrow samples were obtained from donors undergoing orthopedic procedures at the Affiliated Drum Tower Hospital of Nanjing University Medical School. All procedures involving human samples were approved by the Ethics Committee (approval no.: 2021-197-02), and written informed consent was obtained from all participants.

## Data Availability

The data that support the findings of this study are available in the Supplementary Materials. The raw multi-omics datasets supporting the conclusions of this study can be obtained from the corresponding authors upon reasonable request.
