# Supplementary material for: Trabecular-Like Scaffold Dictates Osteogenesis via Fluid Shear Stress-Induced Metabolic Reprogramming through the CAV1–HIF-1α Axis
Source: Research (Wash D C). 2026 Jun 16;9:1307. doi: 10.34133/research.1307 (PMC13270114; doi:10.34133/research.1307)
Supplement: Supplementary 1 — Figs. S1 to S10 Table S1 [file research.1307.f1.zip › Supplementary Materials.docx]

**Supplementary Figure S1.** HFSS‑mediated regulation of osteogenic differentiation in mouse BMSCs.

(A) Western blot analysis of COL1A1, RUNX2, ALP and OPN to detect the effects of HFSS and (B) quantitative of Western blot band intensities. (C) Relative mRNA expression of osteogenic genes determined by qPCR. (D) Representative ALP staining images and corresponding quantitative analysis in static and HFSS groups. Data represent mean ± SD. Statistical significance was assessed using unpaired Student’s t-test.


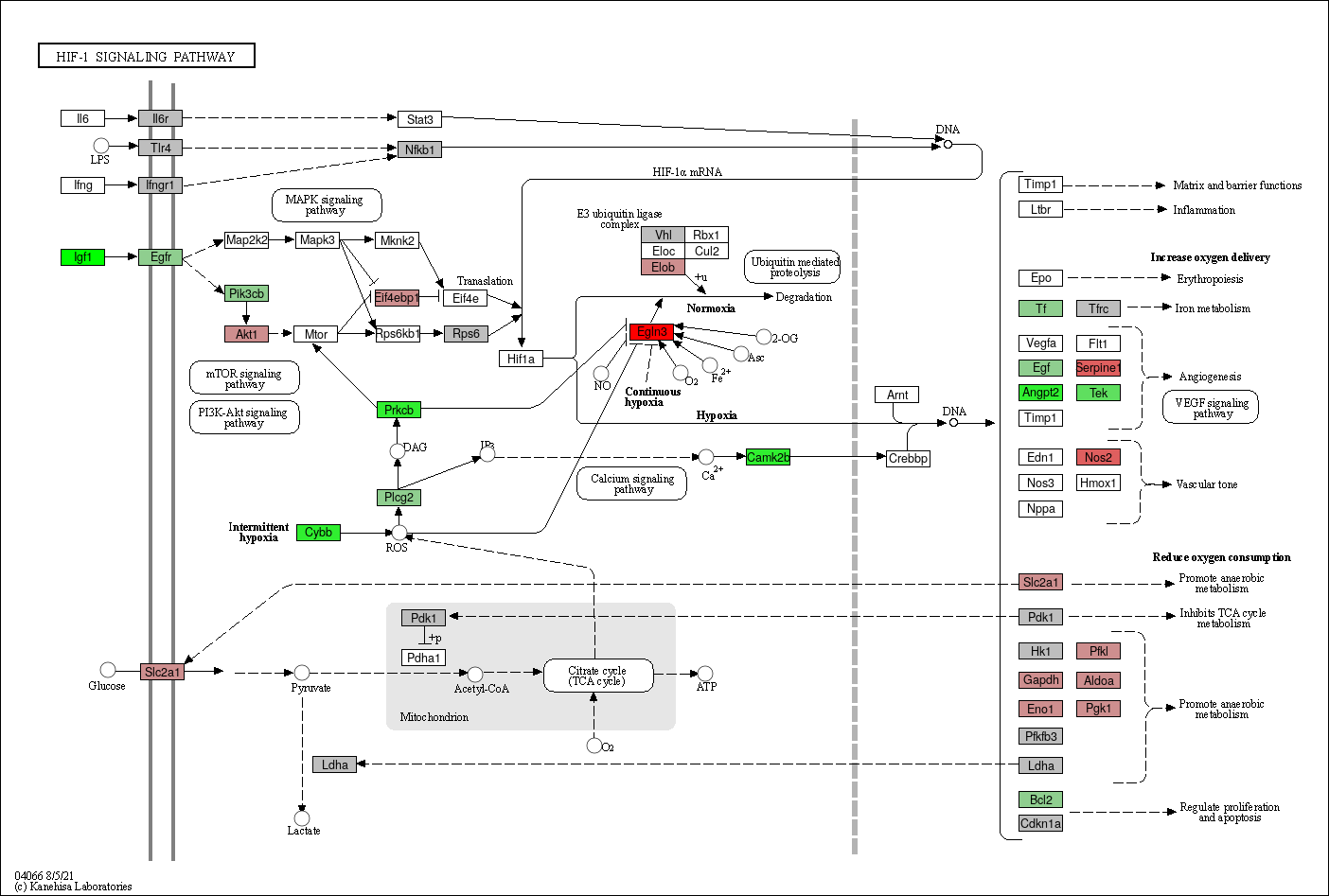


**Supplementary Figure S2.** The gene regulatory network for HIF-1 signaling pathway configured with reference to the KEGG pathway map.


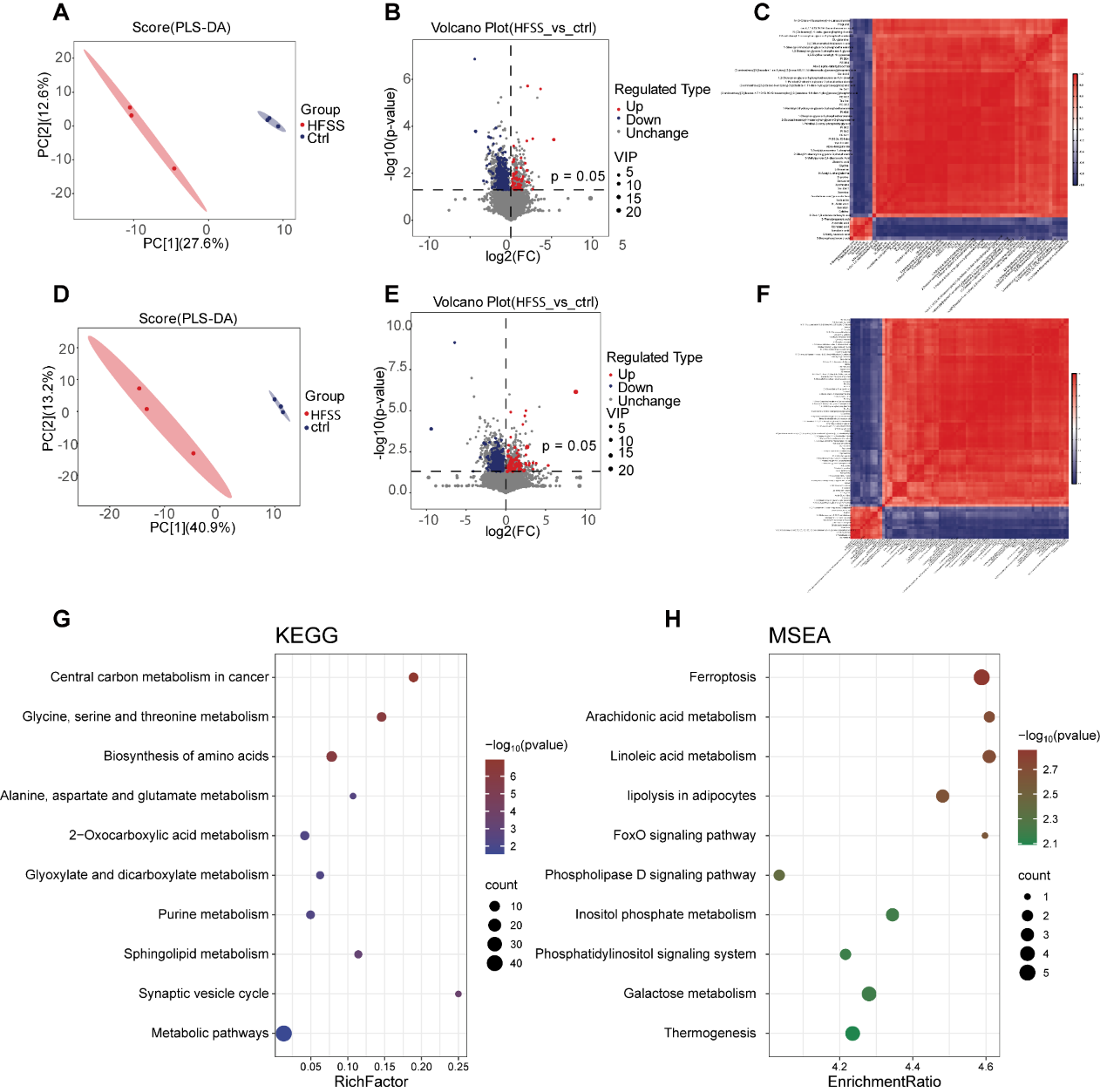


**Supplementary Figure S3.** Global metabolomic profiling of mouse BMSCs under HFSS.

(A) Principal component analysis (PCA) score plot of the negative-ion mode dataset. (B) Volcano plot of negative-ion metabolites comparing HFSS versus Ctrl. (C) Hierarchical clustering and correlation heatmap of significantly altered negative-ion metabolites. (D–F) Corresponding analyses for the positive-ion mode dataset: (D) PCA score plot, (E) volcano plot of differential metabolites and (F) correlation heatmap of significantly altered positive-ion metabolites. (G) KEGG pathway enrichment analysis of differential metabolites. (H) MSEA of the same differential metabolites


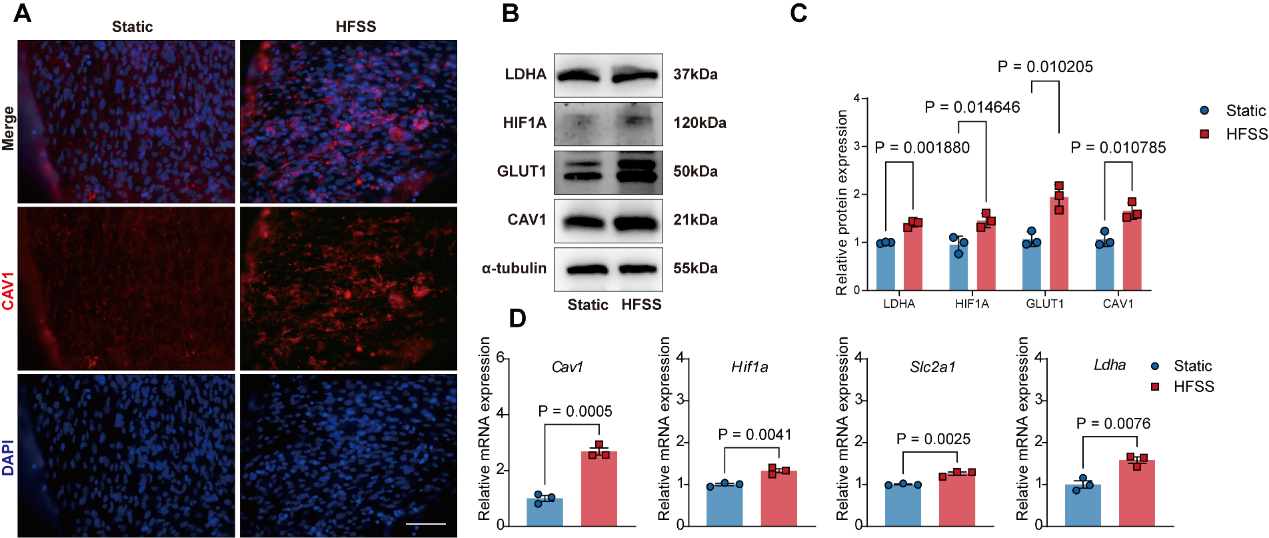


**Supplementary Figure S4.** Validation of integrative omics analysis reveals activation of the CAV1–HIF-1α–glycolysis axis under HFSS conditions.

(A) Representative immunofluorescence images showing CAV1 expression in cells cultured on scaffolds under static and HFSS conditions. Scale bar, 100 μm. (B) Western blot analysis of CAV1, HIF1A, GLUT1, and LDHA protein expression. (C) Quantification of protein expression levels normalized to the loading control. (D) qPCR analysis of *Cav1*, *Hif1a*, *Slc2a1*, and *Ldha* mRNA expression levels. Data represent mean ± SD. Statistical significance was assessed using unpaired Student’s t-test.


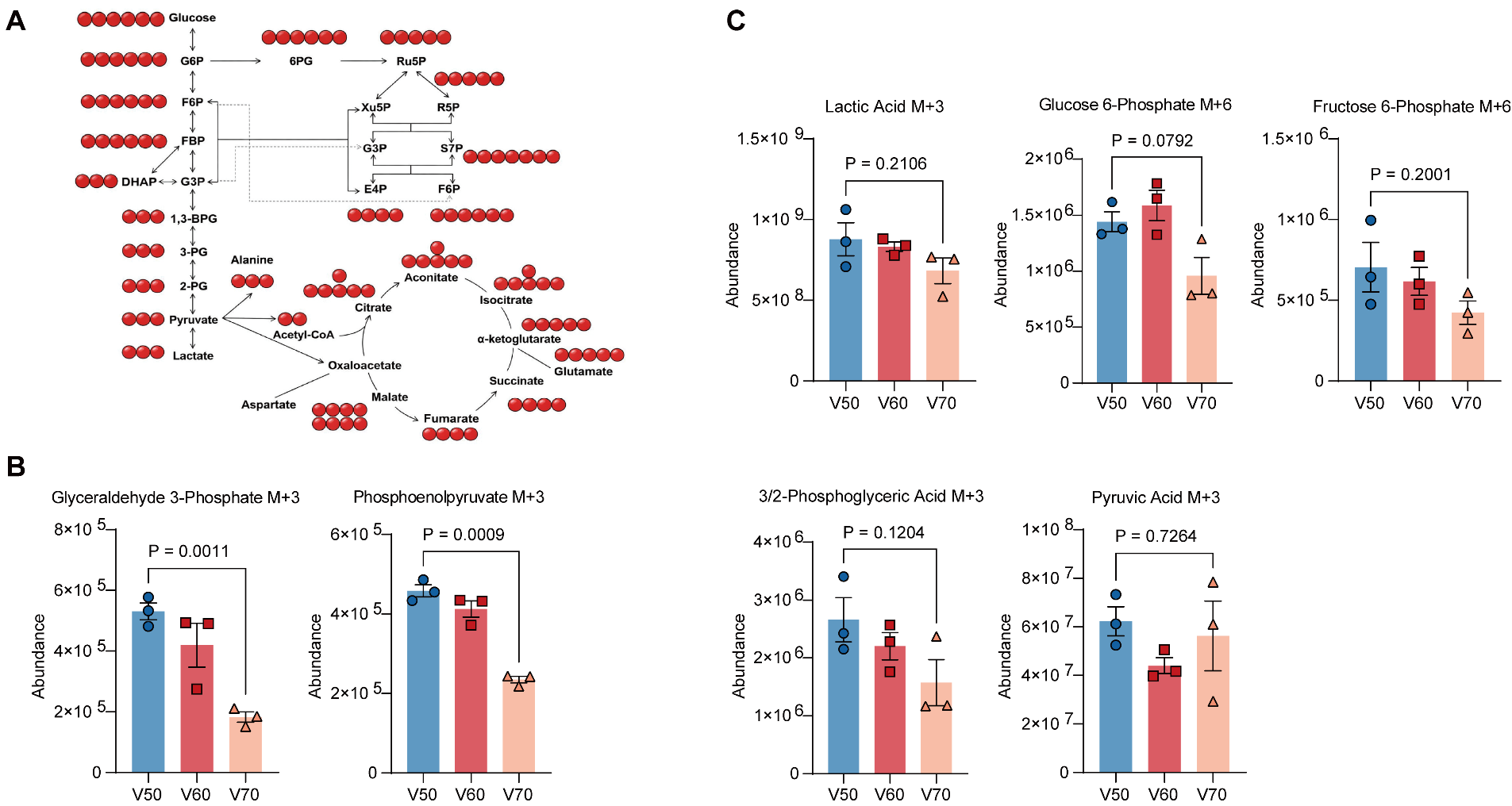


**Supplementary Figure S5.** Supplementary analysis of ^13C_6_-glucose tracing reveals selective changes in labeled glycolytic metabolite abundance under HFSS.

(A) Schematic illustration of ^13C_6_-glucose tracing and the expected incorporation of glucose-derived ^13C into glycolytic intermediates. (B) Quantitative analysis of the abundance of glyceraldehyde-3-phosphate M+3 and phosphoenolpyruvate M+3.

(C) Quantitative analysis of the abundance of other indicated labeled glycolytic metabolites showing no significant differences between groups. Data are presented as mean ± SD (n = 3). Statistical analysis was performed using multiple unpaired two-tailed Welch’s t-tests with Holm–Sidak correction for multiple comparisons.


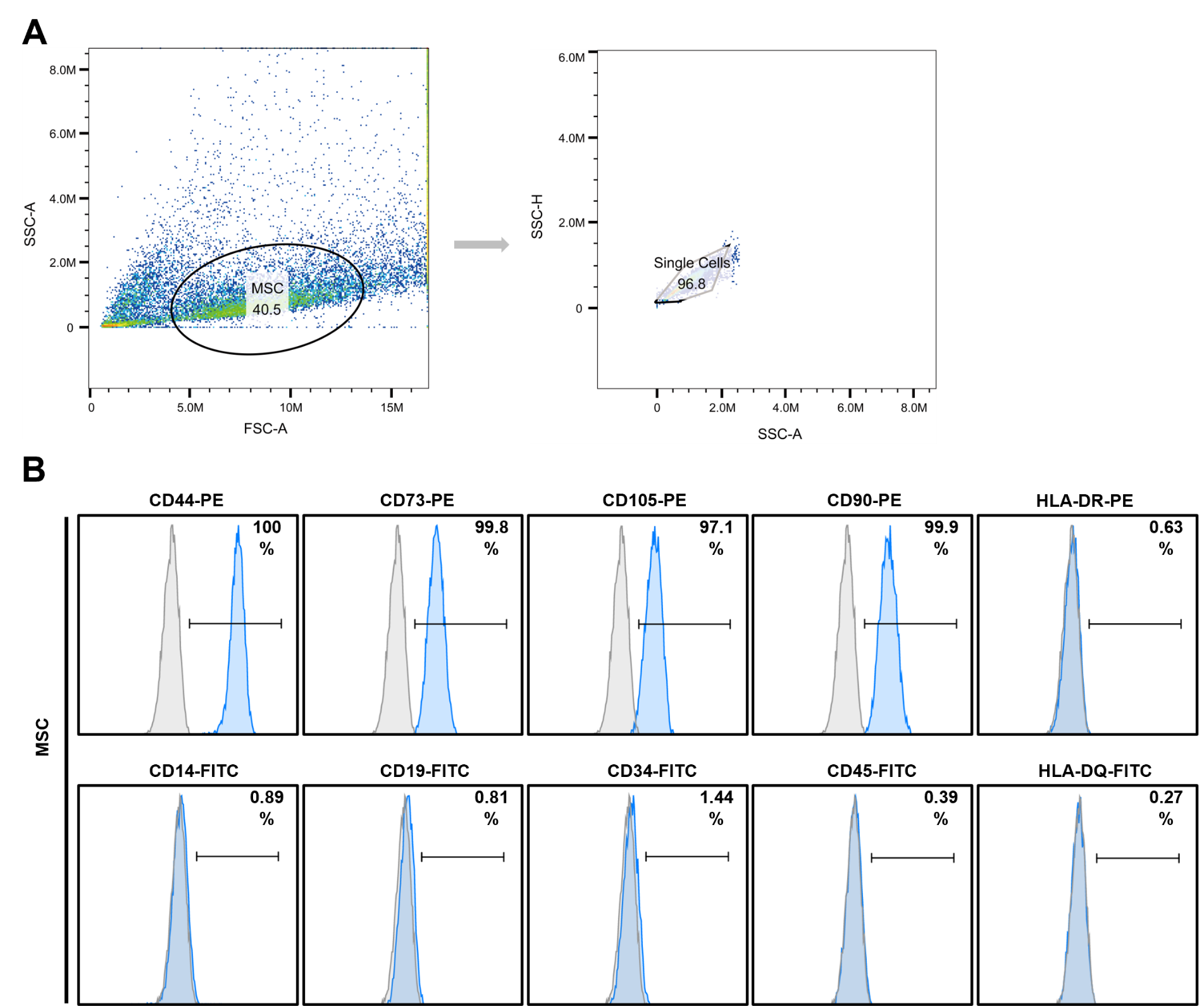


**Supplementary Figure S6.** Flow cytometric characterization of human BMSCs.

(A) Gating strategy used for flow cytometric analysis, including exclusion of debris and doublets, followed by selection of single-cell populations. (B) Representative histograms of surface marker expression. hBMSCs exhibited positive expression of CD44, CD73, CD90, and CD105, and lacked expression of hematopoietic and immune markers including CD14, CD19, CD34, CD45, HLA-DR, and HLA-DQ, consistent with the minimal criteria for defining mesenchymal stem cells.


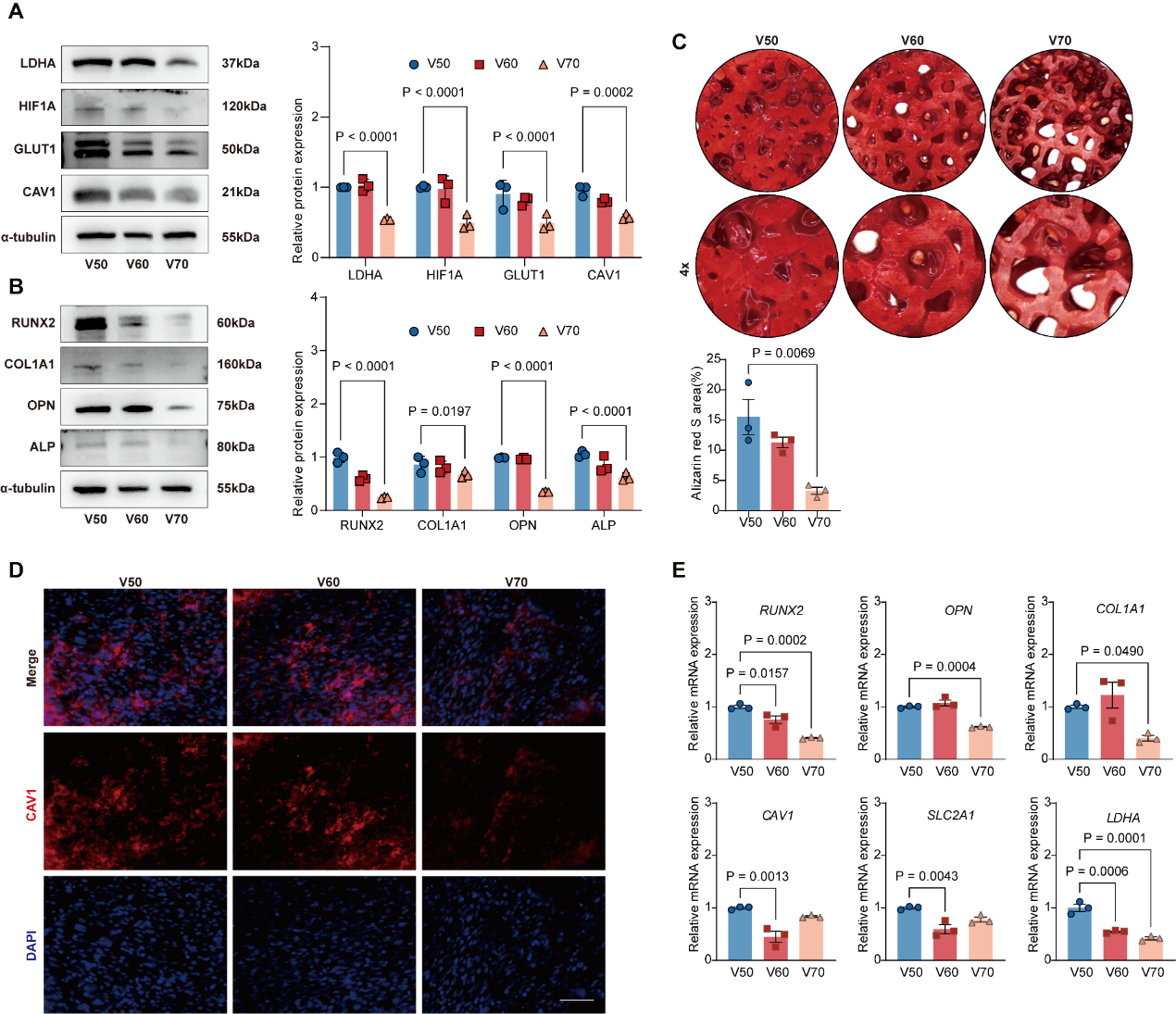


**Supplementary Figure S7.** Human BMSCs cultured on scaffolds recapitulate the mechanometabolic and osteogenic trends.

(A) Western blot analysis and quantification of CAV1, HIF1A, GLUT1, and LDHA expression in human BMSCs cultured on V50, V60, and V70 scaffolds. (B) Western blot analysis and quantification of osteogenic markers. (C) Representative Alizarin Red S staining images and quantitative analysis of mineralized matrix formation after 21 days of osteogenic induction. (D) Representative immunofluorescence images showing CAV1 expression in human BMSCs cultured on the three scaffold types. Scale bar, 100 μm. (E) qPCR analysis of *RUNX2, OPN, COL1A1, CAV1, SLC2A1*, and *LDHA* in human BMSCs. Data are presented as mean ± SD (n = 3). Statistical analysis was performed using one-way ANOVA followed by Tukey’s multiple comparisons test.


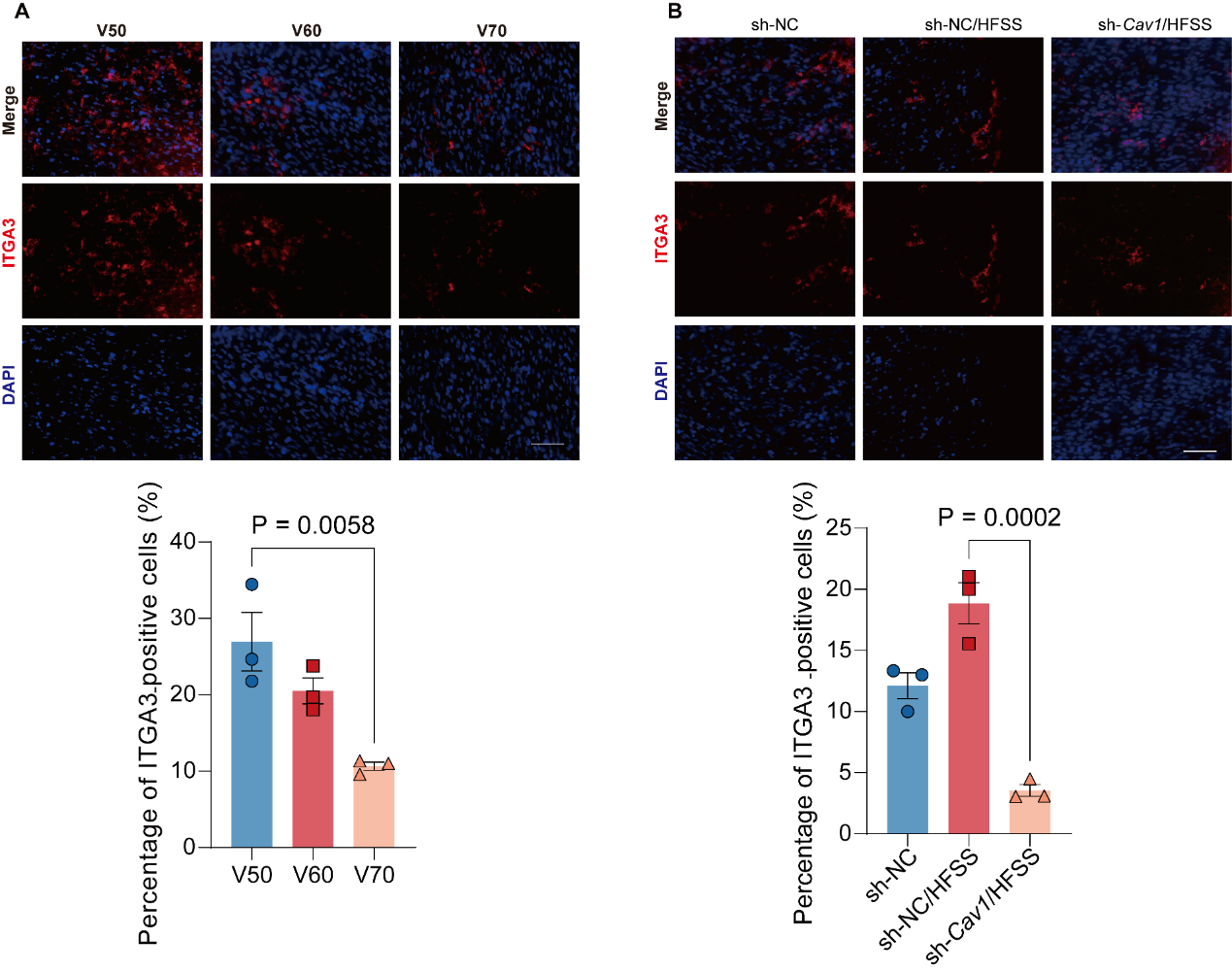


**Supplementary Figure S8.** ITGA3 expression across scaffold conditions and CAV1 modulation.

(A) Representative immunofluorescence images and quantitative analysis of ITGA3 expression in BMSCs cultured on V50, V60, and V70 scaffolds. (B) Representative immunofluorescence images and quantitative analysis of ITGA3 expression in BMSCs under sh-NC, sh-NC/HFSS, and sh-*Cav1*/HFSS conditions. Data are presented as mean ± SD (n = 3). Statistical analysis was performed using one-way ANOVA followed by Tukey’s multiple comparisons test.


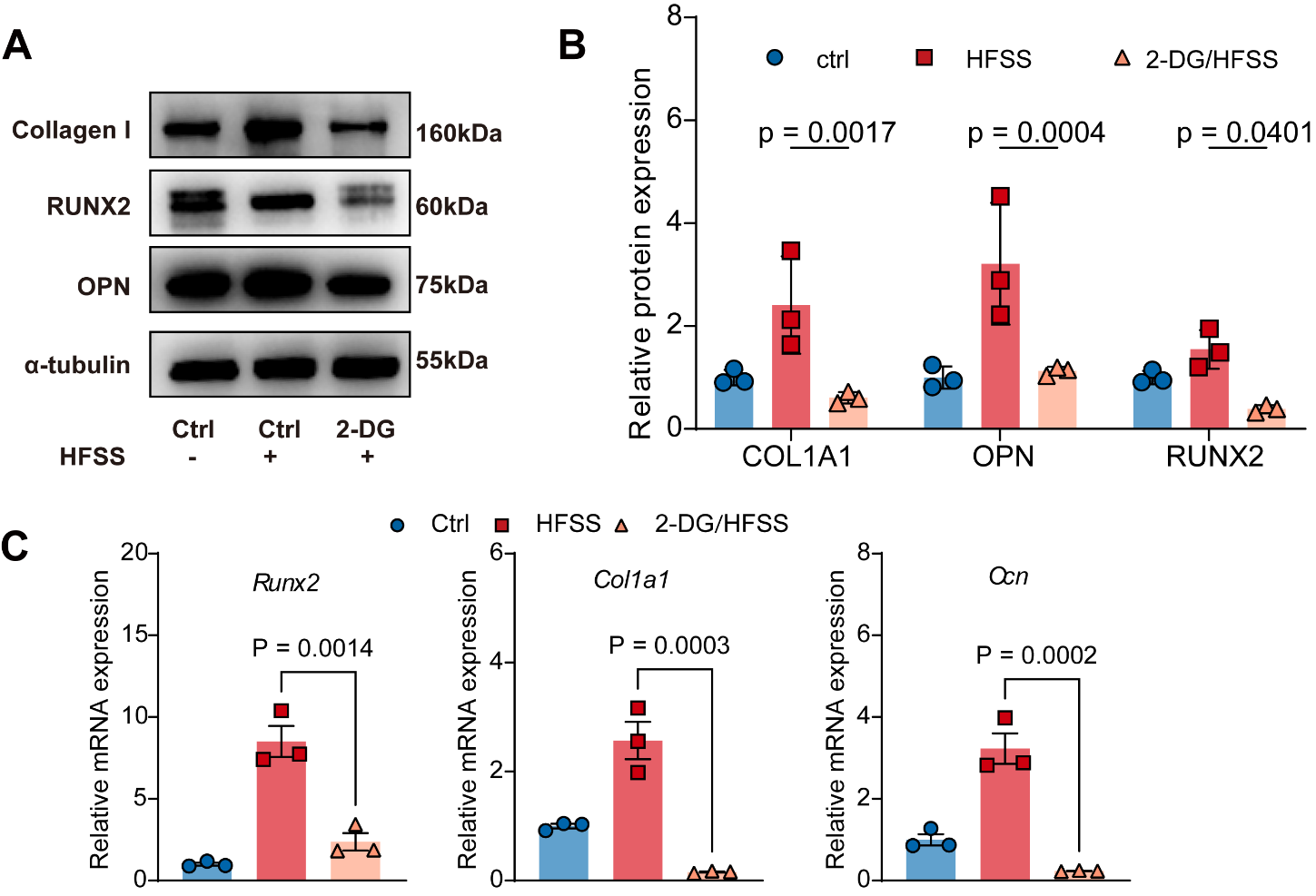


**Supplementary Figure S9.** The HFSS-driven osteogenesis in BMSCs can be inhibited by the glycolysis inhibitor 2-DG.

(A) Western blot analysis of COL1A1, RUNX2 and OPN to detect the effects of 2-DG in blocking glycolysis and (B) quantitative of Western blot band intensities. (C) qPCR analysis of *Col1a1*, *Runx2* and *Opn* mRNA in the 2-DG-treated groups. Data are presented as mean ± SD (n = 3), one-way ANOVA with Tukey’s HSD post hoc test.


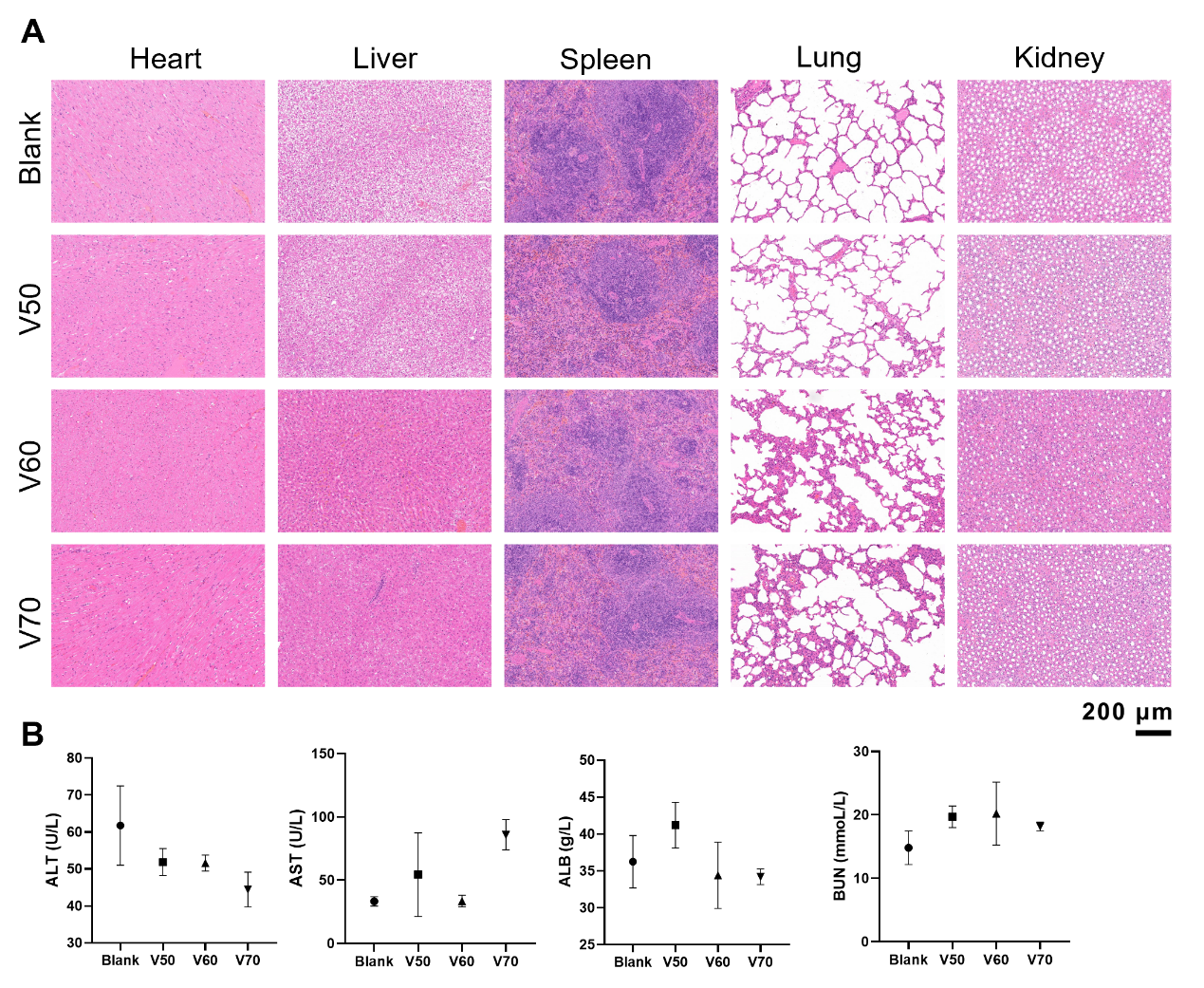


**Supplementary Figure S10.** *In vivo* safety of Voronoi scaffolds.

(A) No significant pathological changes were observed in the heart, liver, spleen, lungs, and kidneys, indicating the biosafety of Voronoi scaffolds for the vital organs of rabbits. (B) Blood chemistry measurements, including alanine aminotransferase (ALT), aspartate aminotransferase (AST), albumin (ALB) and blood urea nitrogen (BUN).

**Supplementary Table S1.** Primer Sequences used for qPCR.

| **Primer** | **Forward(5’-3’)** | **Reverse(3’-5’)** |
| --- | --- | --- |
| *Col1a1* | GGGGCAAGACAGTCATCGAATACA | GTGGAGGGAGTTTACACGAAGCAG |
| *Ocn* | GGACCATCTTTCTGCTCACTCTG | GTTCACTACCTTATTGCCCTCCTG |
| *Opn* | ACAGCCTGCACCCAGATCCTATA | CGTCAGATTCATCCGAGTCCACA |
| *Runx2* | TGCACCTACCAGCCTCACCATAC | GACAGCGACTTCATTCGACTTCC |
| *Slc2a1* | CAGTTCGGCTATAACACTGGTG | GCCCCCGACAGAGAAGATG |
| *Pkm2* | GCCGCCTGGACATTGACTC | CCATGAGAGAAATTCAGCCGAG |
| *Hk2* | TGATCGCCTGCTTATTCACGG | AACCGCCTAGAAATCTCCAGA |
| *Ldha* | TGTCTCCAGCAAAGACTACTGT | GACTGTACTTGACAATGTTGGGA |
| *Cav1* | AACAACCTCAACTGCCTACTCAA | CCCTAAATCCCTAAATCTAACTGG |
| *Tubulin* | CGGGCAGTGTTTGTAGACTTGG | CTCCTTGCCAATGGTGTAGTGC |
| *RUNX2* | CAGAGCAACGTGCTCCAAAGTC | GAAGCGTTGCTGTCGGTTCA |
| *OPN* | CTCCATTGACTCGAACGACTC | CAGGTCTGCGAAACTTCTTAGAT |
| *COL1A1* | GAGGGCCAAGACGAAGACATC | CAGATCACGTCATCGCACAAC |
| *CAV1* | CGCGACCCTAAACACCTCAA | GCCGTCAAAACTGTGTGTCC |
| *SLC2A1* | CTGCAACGGCTTAGACTTCGAC | TCTCTGGGTAACAGGGATCAAACA |
| *LDHA* | TTGACCTACGTGGCTTGGAAG | GGTAACGGAATCGGGCTGAAT |
| *TUBA1B* | GAGGAGATGACTCCTTCAACACC | TGATGAGCTGCTCAGGGTGGAA |
