# Supplementary material for: Trabecular-Like Scaffold Dictates Osteogenesis via Fluid Shear Stress-Induced Metabolic Reprogramming through the CAV1–HIF-1α Axis
Source: Research (Wash D C). 2026 Jun 16;9:1307. doi: 10.34133/research.1307 (PMC13270114; doi:10.34133/research.1307)
Supplement: Supplementary 1 — Figs. S1 to S10 Table S1 [file research.1307.f1.zip › Table S1.docx]

**Supplementary Table S1.** Primer Sequences used for qPCR.

| **Primer** | **Forward(5’-3’)** | **Reverse(3’-5’)** |
| --- | --- | --- |
| *Col1a1* | GGGGCAAGACAGTCATCGAATACA | GTGGAGGGAGTTTACACGAAGCAG |
| *Ocn* | GGACCATCTTTCTGCTCACTCTG | GTTCACTACCTTATTGCCCTCCTG |
| *Opn* | ACAGCCTGCACCCAGATCCTATA | CGTCAGATTCATCCGAGTCCACA |
| *Runx2* | TGCACCTACCAGCCTCACCATAC | GACAGCGACTTCATTCGACTTCC |
| *Slc2a1* | CAGTTCGGCTATAACACTGGTG | GCCCCCGACAGAGAAGATG |
| *Pkm2* | GCCGCCTGGACATTGACTC | CCATGAGAGAAATTCAGCCGAG |
| *Hk2* | TGATCGCCTGCTTATTCACGG | AACCGCCTAGAAATCTCCAGA |
| *Ldha* | TGTCTCCAGCAAAGACTACTGT | GACTGTACTTGACAATGTTGGGA |
| *Cav1* | AACAACCTCAACTGCCTACTCAA | CCCTAAATCCCTAAATCTAACTGG |
| *Tubulin* | CGGGCAGTGTTTGTAGACTTGG | CTCCTTGCCAATGGTGTAGTGC |
| *RUNX2* | CAGAGCAACGTGCTCCAAAGTC | GAAGCGTTGCTGTCGGTTCA |
| *OPN* | CTCCATTGACTCGAACGACTC | CAGGTCTGCGAAACTTCTTAGAT |
| *COL1A1* | GAGGGCCAAGACGAAGACATC | CAGATCACGTCATCGCACAAC |
| *CAV1* | CGCGACCCTAAACACCTCAA | GCCGTCAAAACTGTGTGTCC |
| *SLC2A1* | CTGCAACGGCTTAGACTTCGAC | TCTCTGGGTAACAGGGATCAAACA |
| *LDHA* | TTGACCTACGTGGCTTGGAAG | GGTAACGGAATCGGGCTGAAT |
| *TUBA1B* | GAGGAGATGACTCCTTCAACACC | TGATGAGCTGCTCAGGGTGGAA |
