# Supplementary figures and images for: Trabecular-Like Scaffold Dictates Osteogenesis via Fluid Shear Stress-Induced Metabolic Reprogramming through the CAV1–HIF-1α Axis
Source: Research (Wash D C). 2026 Jun 16;9:1307. doi: 10.34133/research.1307 (PMC13270114; doi:10.34133/research.1307)

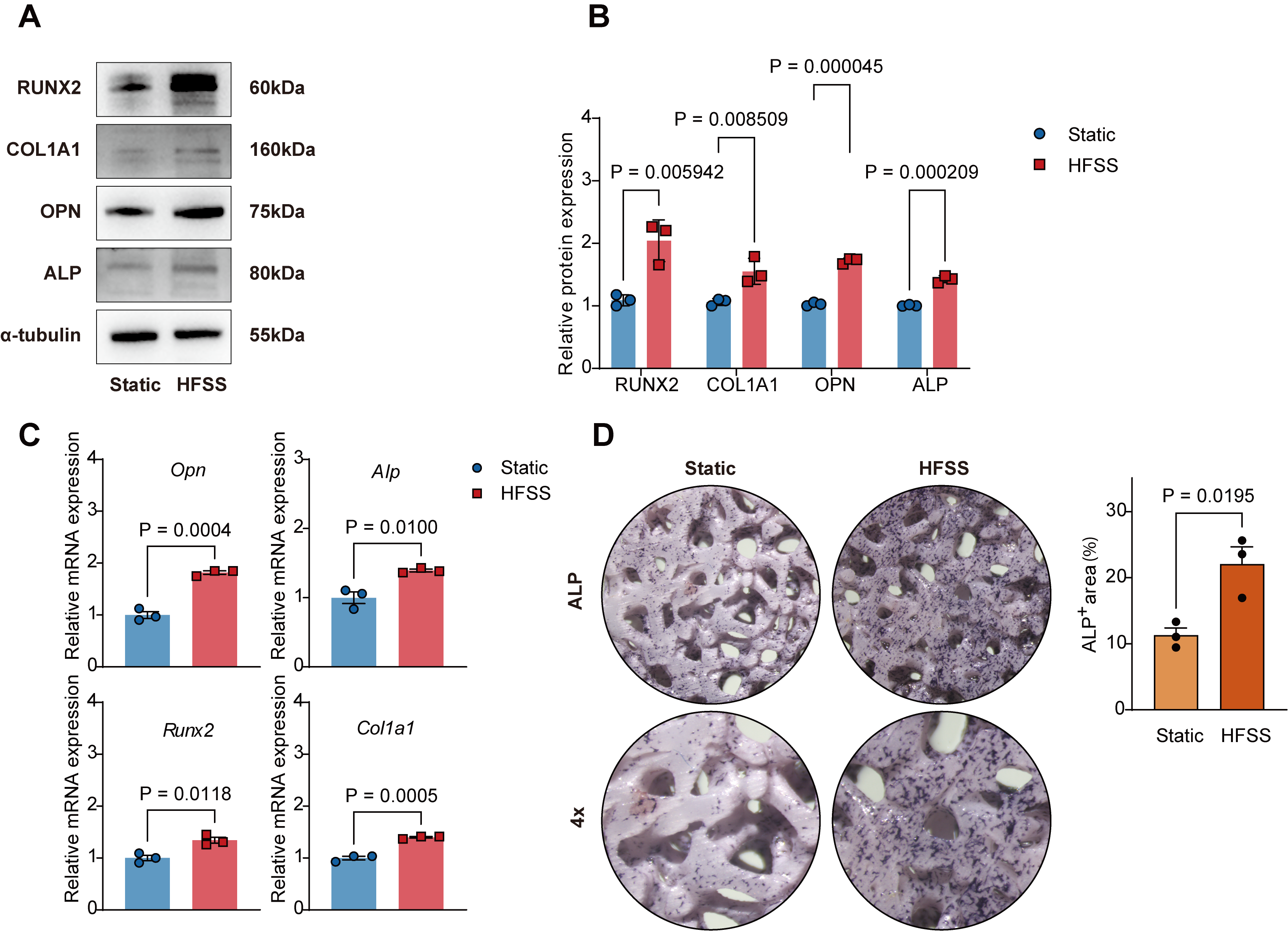

Supplement: Supplementary 1 — Figs. S1 to S10 Table S1 [file research.1307.f1.zip › Figure S1.tif]

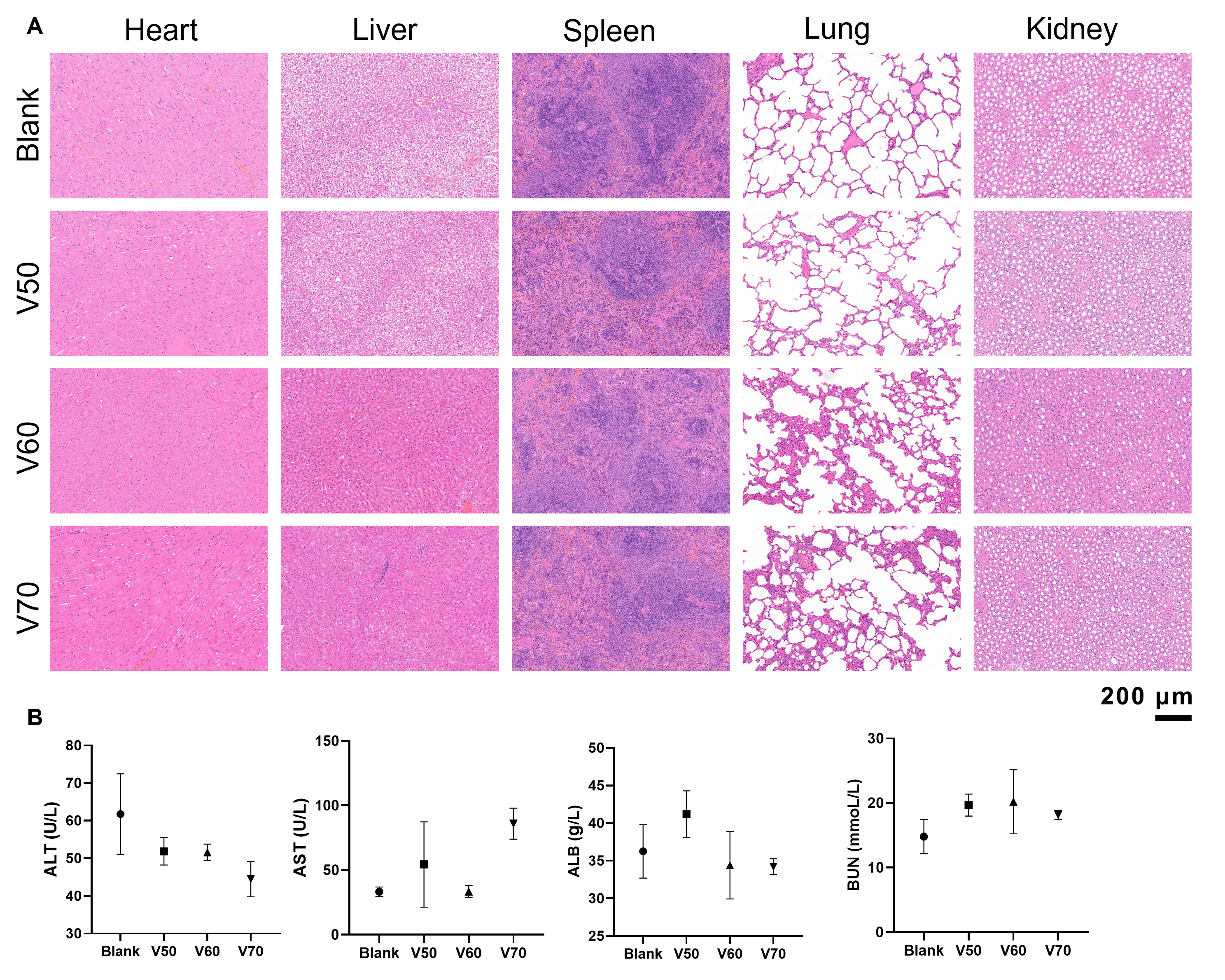

Supplement: Supplementary 1 — Figs. S1 to S10 Table S1 [file research.1307.f1.zip › Figure S10.tif]

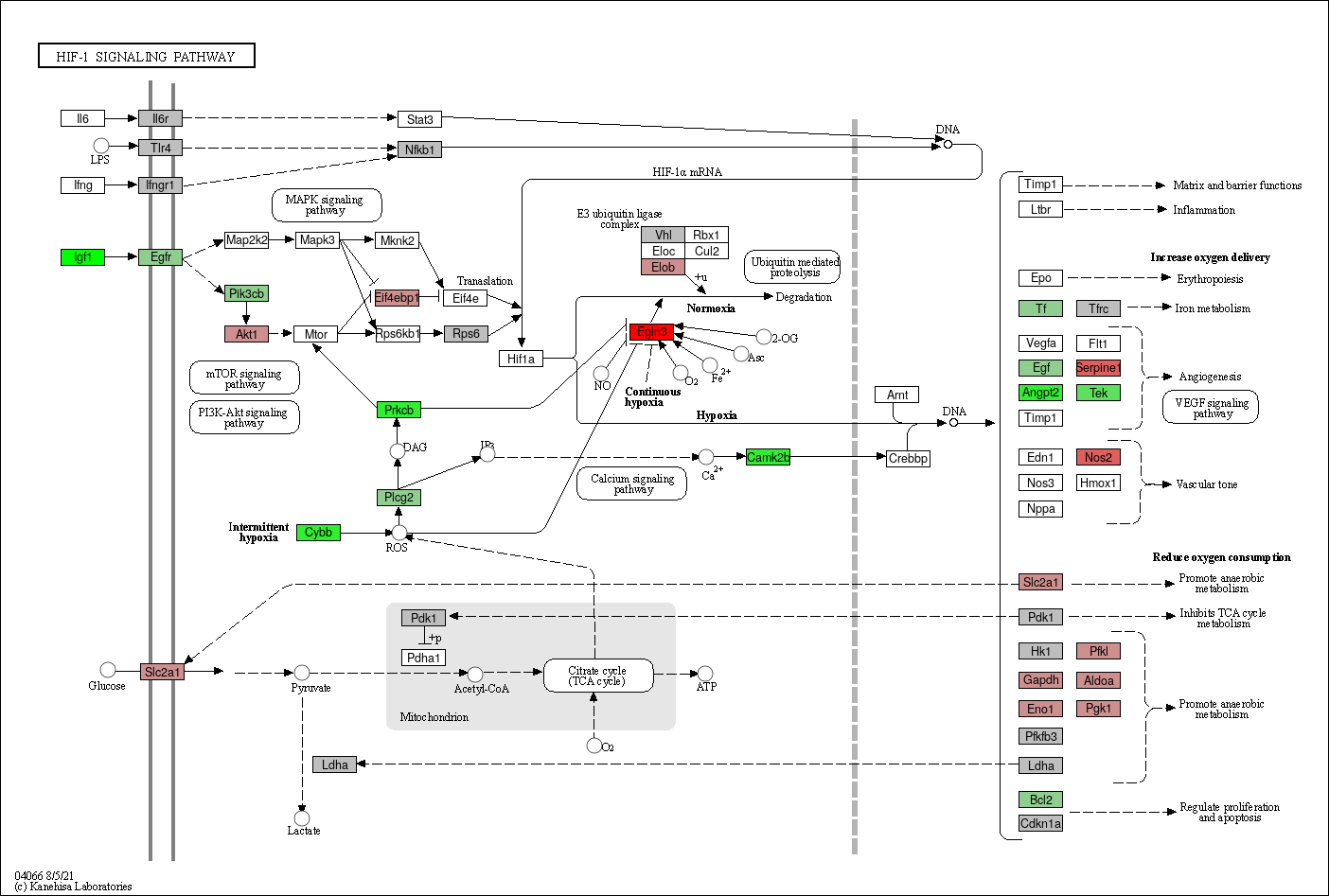

Supplement: Supplementary 1 — Figs. S1 to S10 Table S1 [file research.1307.f1.zip › Figure S2.png]

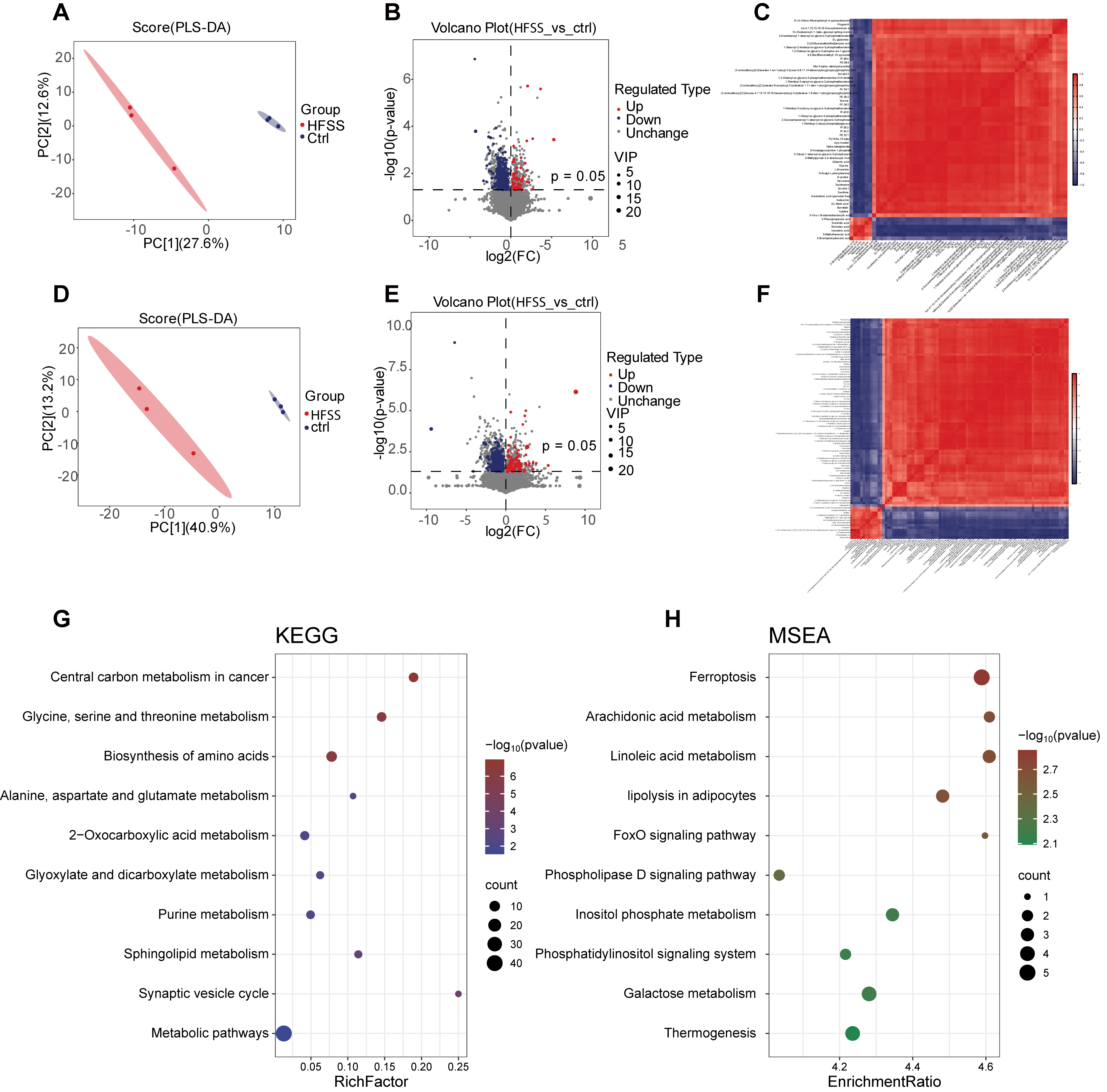

Supplement: Supplementary 1 — Figs. S1 to S10 Table S1 [file research.1307.f1.zip › Figure S3.tif]

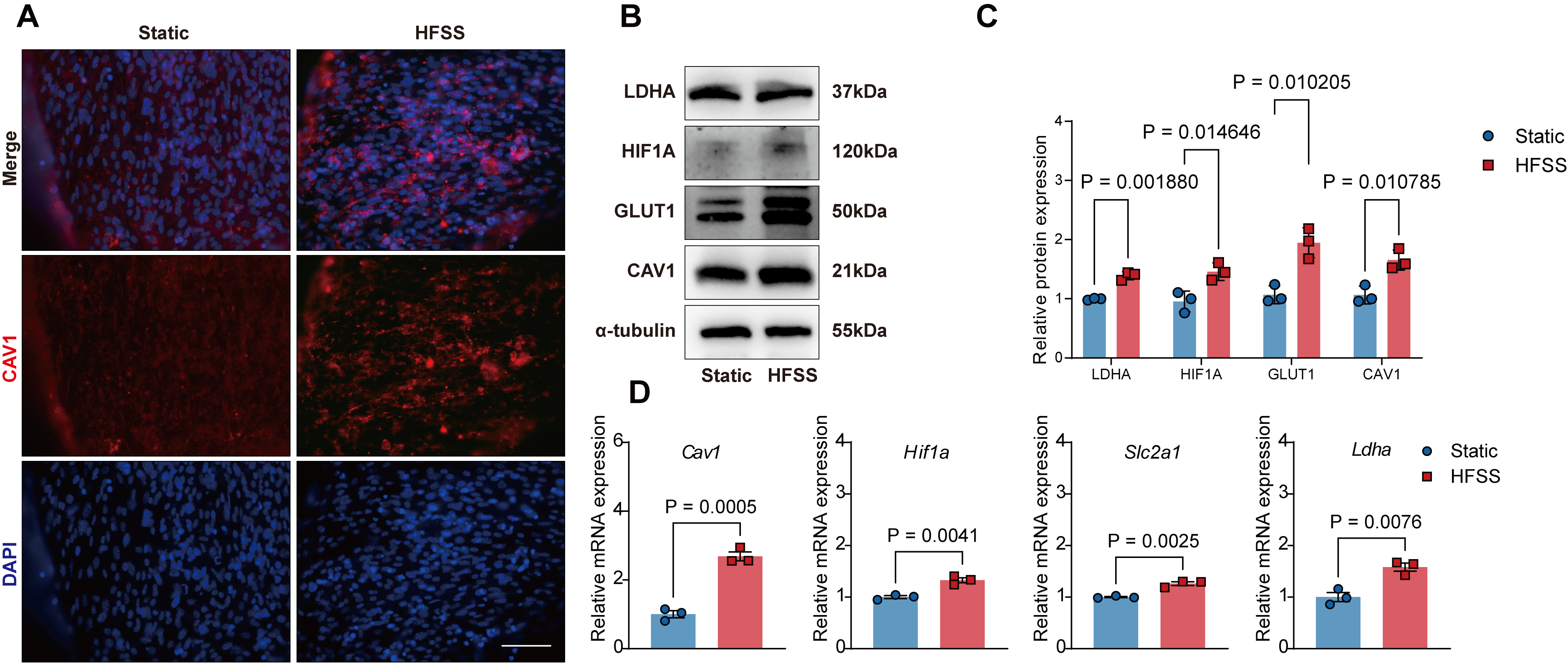

Supplement: Supplementary 1 — Figs. S1 to S10 Table S1 [file research.1307.f1.zip › Figure S4.tif]

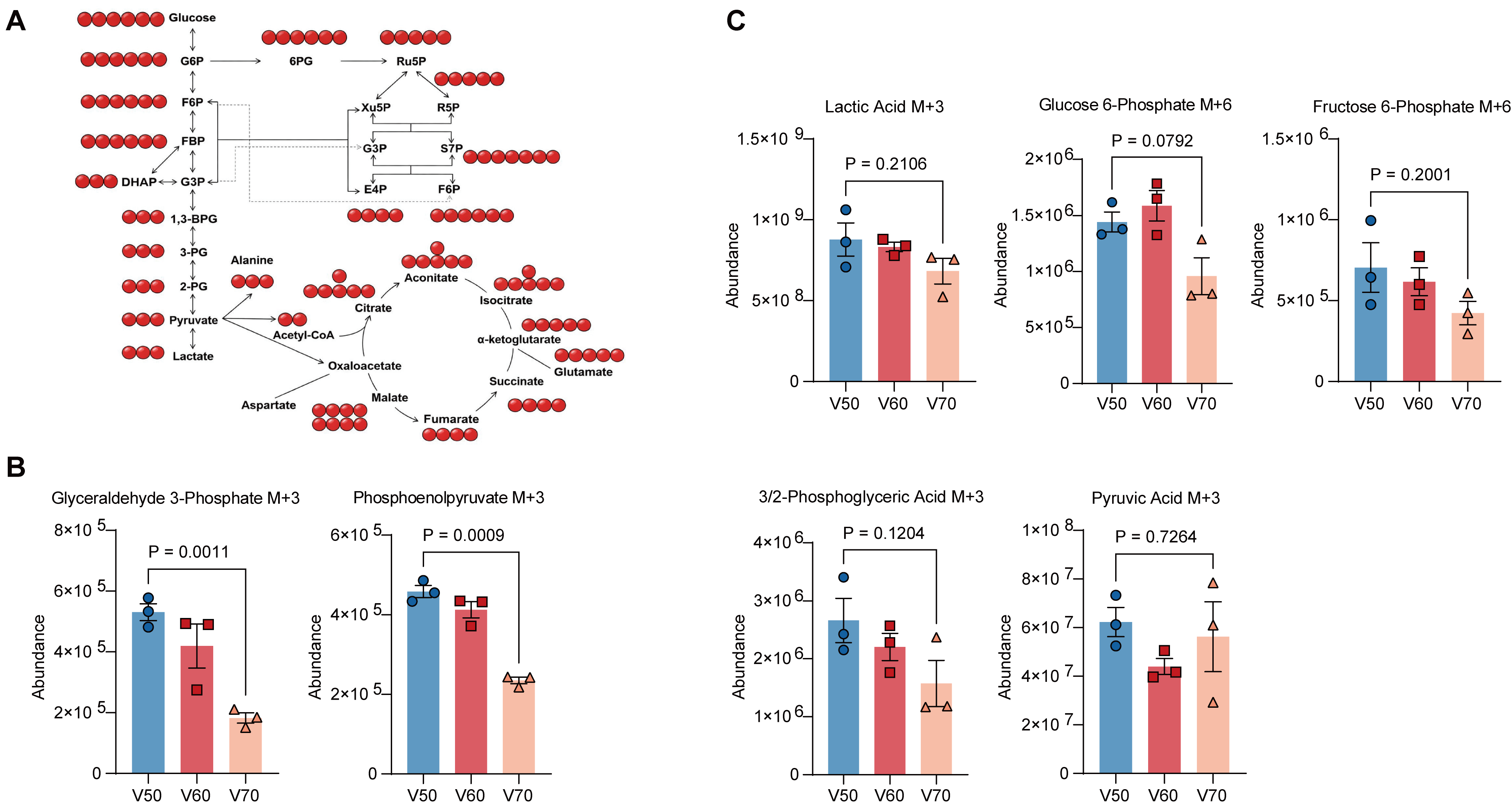

Supplement: Supplementary 1 — Figs. S1 to S10 Table S1 [file research.1307.f1.zip › Figure S5.tif]

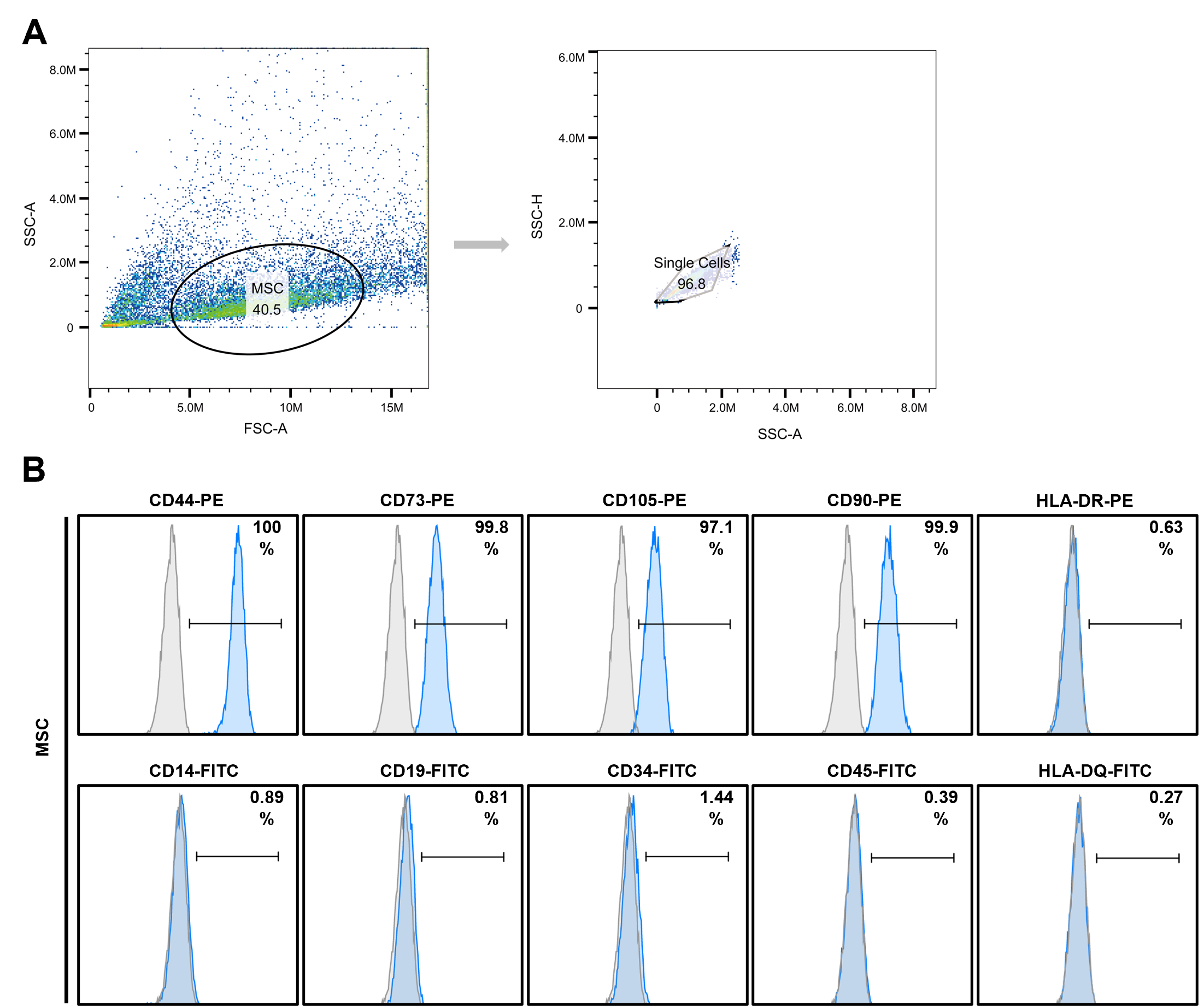

Supplement: Supplementary 1 — Figs. S1 to S10 Table S1 [file research.1307.f1.zip › Figure S6.tif]

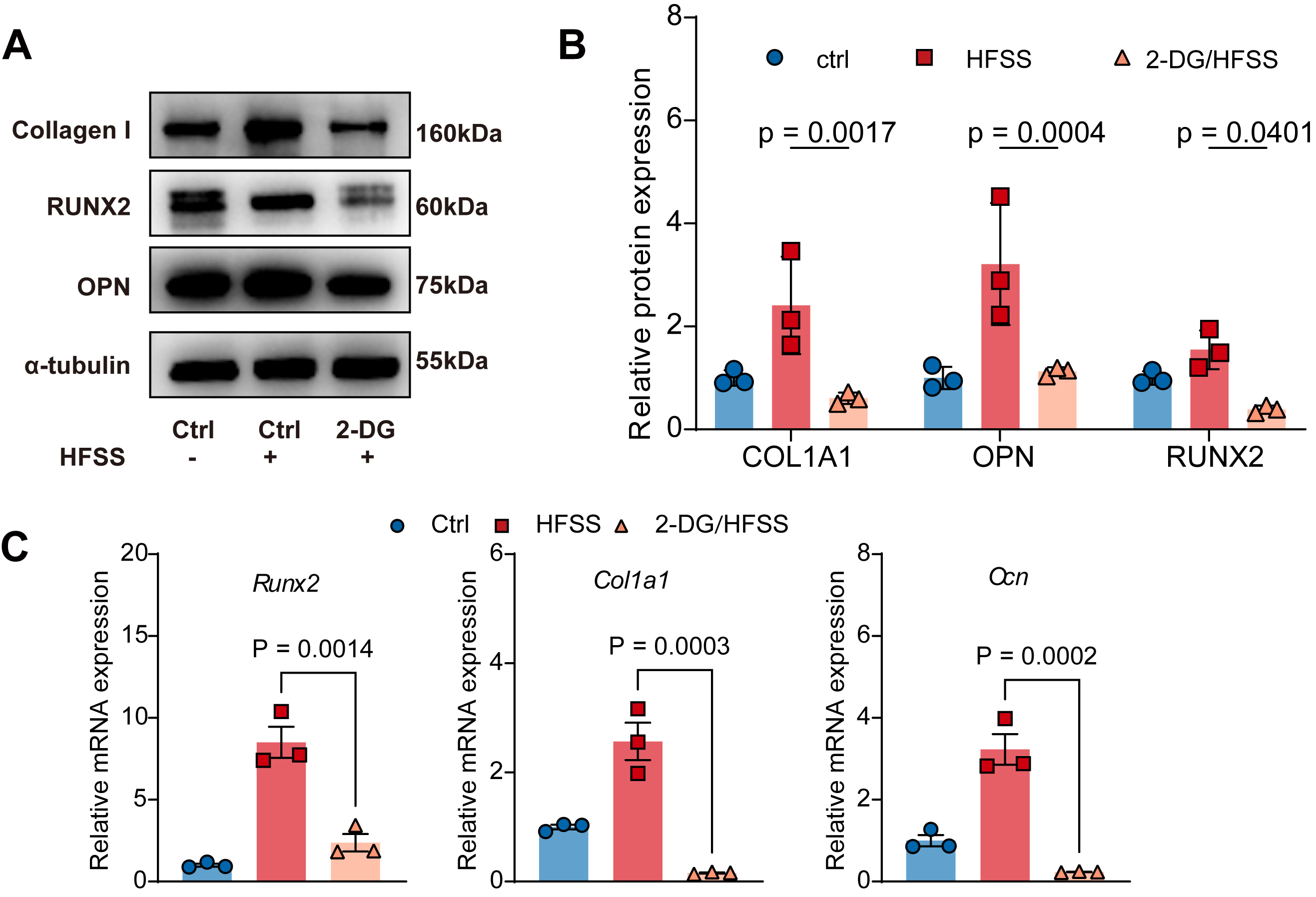

Supplement: Supplementary 1 — Figs. S1 to S10 Table S1 [file research.1307.f1.zip › Figure S9.tif]
